# Immune-interacting lymphatic endothelial subtype at capillary terminals drives lymphatic malformation

**DOI:** 10.1084/jem.20220741

**Published:** 2023-01-23

**Authors:** Milena Petkova, Marle Kraft, Simon Stritt, Ines Martinez-Corral, Henrik Ortsäter, Michael Vanlandewijck, Bojana Jakic, Eulàlia Baselga, Sandra D. Castillo, Mariona Graupera, Christer Betsholtz, Taija Mäkinen

**Affiliations:** 1 Department of Immunology, Genetics and Pathology, Uppsala University, Uppsala, Sweden; 2 Department of Medicine Huddinge, Karolinska Institutet, Campus Flemingsberg, Neo, Huddinge, Sweden; 3 Department of Dermatology, Hospital Sant Joan de Déu, Esplugues de Llobregat, Spain; 4 Endothelial Pathobiology and Microenvironment Group, Josep Carreras Leukaemia Research Institute (IJC), Badalona, Spain; 5 CIBERONC, Instituto de Salud Carlos III, Madrid, Spain; 6 ICREA, Barcelona, Spain

## Abstract

Oncogenic mutations in *PIK3CA*, encoding p110α-PI3K, are a common cause of venous and lymphatic malformations. Vessel type–specific disease pathogenesis is poorly understood, hampering development of efficient therapies. Here, we reveal a new immune-interacting subtype of *Ptx3*-positive dermal lymphatic capillary endothelial cells (iLECs) that recruit pro-lymphangiogenic macrophages to promote progressive lymphatic overgrowth. Mouse model of *Pik3ca*^*H1047R*^-driven vascular malformations showed that proliferation was induced in both venous and lymphatic ECs but sustained selectively in LECs of advanced lesions. Single-cell transcriptomics identified the iLEC population, residing at lymphatic capillary terminals of normal vasculature, that was expanded in *Pik3ca*^*H1047R*^ mice. Expression of pro-inflammatory genes, including monocyte/macrophage chemokine *Ccl2*, in *Pik3ca*^*H1047R*^-iLECs was associated with recruitment of VEGF-C–producing macrophages. Macrophage depletion, CCL2 blockade, or anti-inflammatory COX-2 inhibition limited *Pik3ca*^*H1047R*^-driven lymphangiogenesis. Thus, targeting the paracrine crosstalk involving iLECs and macrophages provides a new therapeutic opportunity for lymphatic malformations. Identification of iLECs further indicates that peripheral lymphatic vessels not only respond to but also actively orchestrate inflammatory processes.

## Teaser

Petkova et al. identify a new *Ptx3*-positive immune-interacting subtype of lymphatic endothelial cells, iLECs, that drive oncogenic PI3K-driven lymphatic malformations. These cells produce chemokines that recruit pro-lymphangiogenic macrophages, which in turn promote pathological lymphatic vessel growth.

## Graphical Abstract

**Figure:**
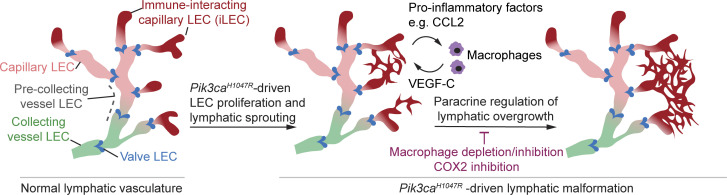

## Introduction

Venous malformations (VMs) and lymphatic malformations (LMs) are chronic diseases characterized by vascular lesions that range from simple skin discoloration to large deformations, or fluid-filled cysts to infiltrative soft-tissue masses, respectively (Queisser et al., 2021; Mäkinen et al., 2021). They are often associated with significant morbidity, and in some cases life-threatening complications, due to pain, bleeding, and functional impairment of nearby organs. Somatic-activating *PIK3CA* mutations have been identified as causative of the majority of LMs (Luks et al., 2015; Osborn et al., 2015) and a smaller proportion of VMs (Limaye et al., 2015; Castel et al., 2016; Castillo et al., 2016). *PIK3CA* is frequently mutated also in cancer and other pathologies characterized by tissue hyperplasia, the so-called PIK3CA-related overgrowth spectrum (Madsen et al., 2018; Angulo-Urarte and Graupera, 2022).

*PIK3CA* encodes the p110α subunit of the phosphoinositide 3-kinase (PI3K) that catalyzes the production of phosphatidylinositol (3,4,5)-triphosphate (PIP_3_) at the plasma membrane, leading to activation of downstream signaling cascades such as the AKT-mTOR (mammalian target of rapamycin) pathway. PI3K signaling controls a variety of cellular processes in both blood and lymphatic vasculatures, including endothelial cell (EC) migration, survival, and proliferation as well as vessel sprouting, thereby critically regulating vascular maintenance and growth (Angulo-Urarte and Graupera, 2022). Genetic loss-of-function studies in mice have uncovered a critical role of p110α in the normal development of blood and lymphatic vessels (Graupera et al., 2008; Gupta et al., 2007; Stanczuk et al., 2015). Conversely, the expression of an activating *PIK3CA* mutation in ECs led to vascular overgrowth and malformations in mice (di Blasio et al., 2018; Rodriguez-Laguna et al., 2019; Martinez-Corral et al., 2020). EC-autonomous effects in the pathogenesis of both VM and LM are demonstrated by the presence of *PIK3CA* mutations specifically in ECs but not in other cell types (Osborn et al., 2015; Blesinger et al., 2018; Boscolo et al., 2015).

*PIK3CA* mutations frequently occur in two hot spot regions encoding the helical domain and the kinase domain, with an H1047R substitution in the latter representing one of the most frequent VM/LM and cancer mutation (Madsen et al., 2018; Queisser et al., 2021; Mäkinen et al., 2021). Identification of PIK3CA mutations as drivers of vascular malformations has enabled repurposing available in Food and Drug Administration–approved inhibitors of the PI3K pathway for their treatment. For example, rapamycin that targets the PI3K-AKT downstream effector mTOR has shown efficacy in relieving symptoms in VM and LM patients although it rarely results in the regression of lesions (Queisser et al., 2021; Mäkinen et al., 2021). Apart from the identified EC-autonomous mutations driving vascular anomalies, emerging evidence points to synergistically acting paracrine mechanisms that contribute to disease progression (Mäkinen et al., 2021). For example, increased paracrine vascular endothelial growth factor C (VEGF-C) signaling is observed in LMs in mice and human patients (Boscolo et al., 2015; Martinez-Corral et al., 2020; Partanen et al., 2013), and it is required for the growth of *Pik3ca*-driven LM in mice (Martinez-Corral et al., 2020). Interestingly, the inhibition of VEGF-C was more effective than rapamycin in limiting LM growth in mice, and when administered in combination with rapamycin, it even promoted regression of the abnormal lymphatic vessels (Martinez-Corral et al., 2020). Better understanding of both the aberrant EC-autonomous signaling and the paracrine mechanisms should aid the development of effective and targeted combinatorial therapies for LMs and other vascular malformations.

Here, we investigated the endothelial subtype–specific mechanisms underlying *PIK3CA*-driven LM in comparison with VM. Analyses of mouse models of *Pik3ca*^*H1047R*^-driven vascular malformations revealed lymphatic and blood vessel type–specific responses resulting in distinct lesion characteristics. Selective features of LM were tissue infiltration of myeloid cells producing pro-lymphangiogenic factors during early stages of active vascular growth, which occurred concomitant with an increase in cytokine levels and expansion of an immune-interacting capillary LEC subtype, iLEC, identified through single-cell transcriptomics. Importantly, macrophage depletion, CCL2 blockade, or anti-inflammatory cyclooxygenase-2 (COX-2) inhibition limited *Pik3ca*^*H1047R*^-driven lymphangiogenesis in mice. These results show that paracrine immune activation driven by LEC-autonomous oncogenic p110α-PI3K signaling critically contributes to pathological vascular growth in LM and provides a therapeutic target.

## Results

### Vessel type–specific responses to embryonic activation of oncogenic *Pik3ca*

**Figure 1.**
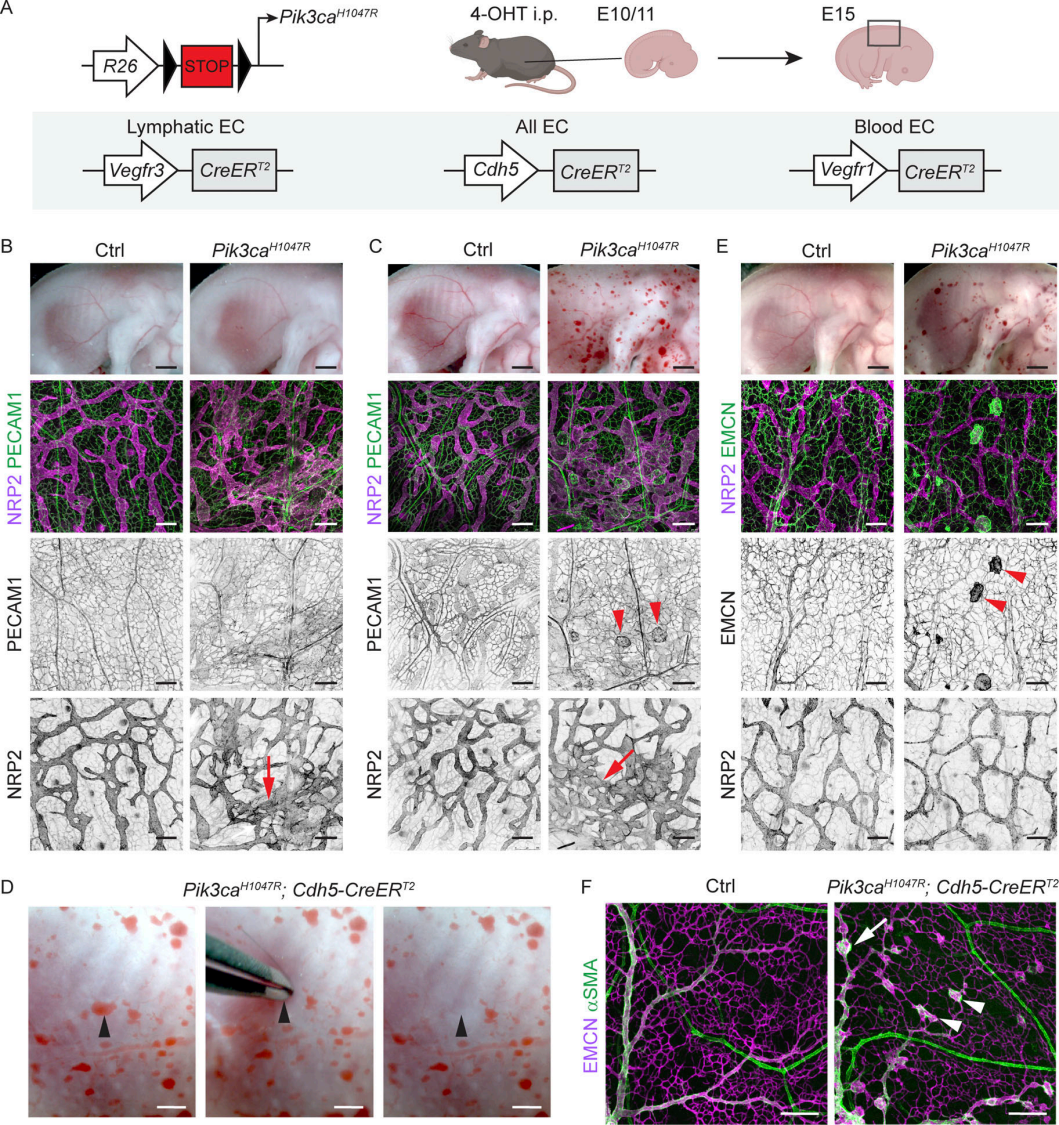
**Vessel type–specific responses to activation of oncogenic *Pik3ca* signaling in the embryonic vasculature. (A)** Genetic constructs and strategy for tamoxifen-inducible *Pik3ca*^*H1047R*^ expression in embryonic lymphatic (*Vegfr3-CreER*^*T2*^), lymphatic and blood (*Cdh5-CreER*^*T2*^), or specifically in blood (*Vegfr1-CreER*^*T2*^) endothelia. **(B and C)** E15 *Pik3ca*^*H1047R*^;*Vegfr3-CreER*^*T2*^ (B), *Pik3ca*^*H1047R*^;*Cdh5-CreER*^*T2*^ (C), and their littermate control (Ctrl) embryos treated with 4-OHT at E11. Whole-mount immunofluorescence of the back skin is shown below. Single channel images show hyperbranching of NRP2^+^ lymphatic vessels (red arrows) in both models, but presence of PECAM1^+^ blood vessel lesions (red arrowheads) only in the *Cdh5-CreER*^*T2*^ model. **(D)** Evacuation of blood upon application of pressure on a blood-filled lesion (arrowhead) in the skin of E15 *Pik3ca*^*H1047R*^;*Cdh5-CreER*^*T2*^ embryo. **(E)** E15 *Pik3ca*^*H1047R*^;*Vegfr1-CreER*^*T2*^ embryos treated with 4-OHT at E10, and whole-mount immunofluorescence of the back skin showing EMCN^+^ lesions (red arrowheads) in the blood vessels and normal lymphatic vasculature. **(F)** Whole-mount immunofluorescence of E17 skin from *Pik3ca*^*H1047R*^;*Cdh5-CreER*^*T2*^ and littermate control (Ctrl) embryos treated with 4-OHT at E14. Note that lesions are present in the EMCN^+^ veins (arrow) and capillaries (arrowheads) but not in the αSMA^+^ arteries. Scale bar: 1 mm (B, C, and E, top panels) 200 µm (B–F).

**Figure S1.**
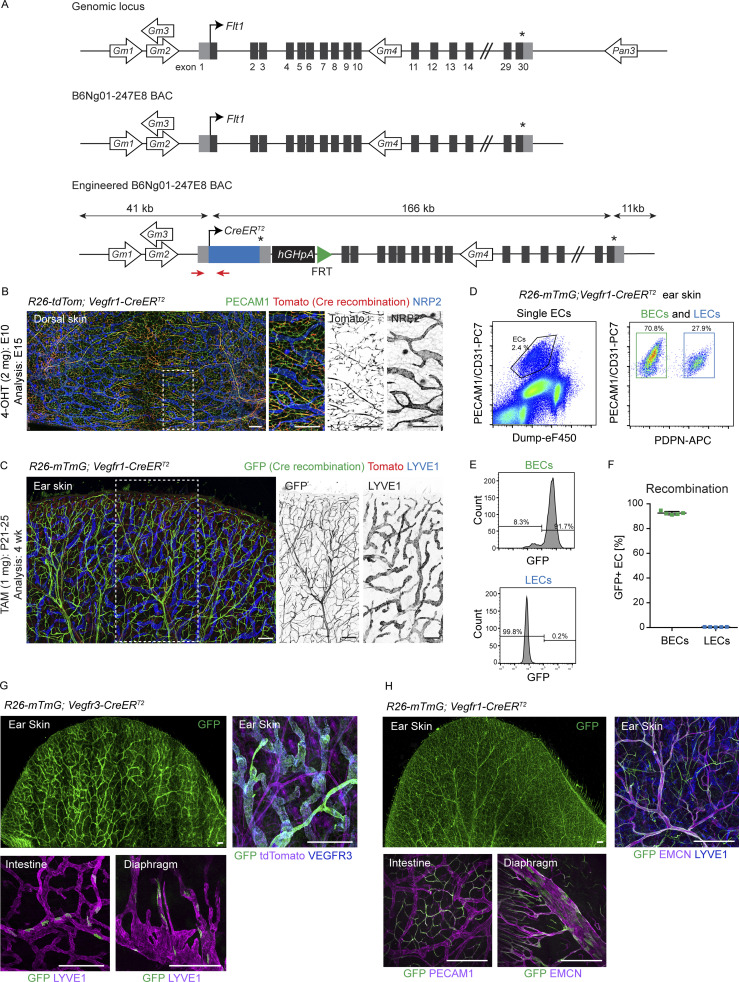
**Generation and characterization of BAC transgenic *Vegfr1-CreER***^***T2***^ **mice. (A)** Schematic of the genomic locus, a BAC for the *Flt1* (*Vegfr1*) gene, and the engineered BAC used for generation of transgenic mice where exon 1 of *Vegfr1* is replaced with the open reading frame for *CreER*^*T2*^, the *Vegfr1* 3′ UTR and hGH polyadenylation signal. In addition to the *Vegfr1* gene, the engineered BAC also carries the *Gm35592* ncRNA (non-coding RNA; NCBI gene ID: 102639236), the *Gm35541* ncRNA (NCBI gene ID: 102639166), the *Gm43156* long intergenic ncRNA (Ensembl gene ID: ENSMUSG00000106845) and the *Gm35383* ncRNA (NCBI gene ID: 102638948). The locations of the genotyping primers are indicated (red arrows). Asterisk indicates stop codon. **(B)** Whole-mount immunofluorescence of the skin of E15 *R26-tdTom*;*Vegfr1-CreER*^*T2*^ embryo showing tdTom expression (Cre-mediated recombination) specifically in the PECAM1^+^LYVE1^−^ blood vessels. Boxed area is magnified on the right. 4-OHT was administered at E10. **(C)** Whole mount immunofluorescence of 4-wk-old *R26-mTmG*;*Vegfr1-CreER*^*T2*^ ear skin showing GFP expression (Cre-mediated recombination) specifically in the LYVE1^−^ blood vessels. Single channel images are shown for the boxed area. **(D–F)** Flow cytometry analysis of dermal ECs from *R26-mTmG*;*Vegfr1-CreER*^*T2*^ ear (D and E) showing efficient recombination (GFP expression) specifically in the BECs. Data in F represent mean (*n* = 5 mice) ± SD. In C–F, mice received five consecutive administrations of 1 mg of tamoxifen at 3 wk of age and were analyzed at 4 wk of age. Percentages of gated cells in the gating scheme: 2.4% ECs, from which 70.8% BECs and 27.9% LECs in D; 91.7% GFP^+^ LECs and 99.8% GFP^−^ BECs in E. **(G and H)** Cre recombination efficiency, visualized by GFP expression, in a whole-mount ear skin and the internal organs of *R26-mTmG*;*Vegfr3-CreER*^*T2*^ (G) and *R26-mTmG*;*Vegfr1-CreER*^*T2*^ (H) mice after topical application of 4-OHT to the ears. Systemic recombination was observed at a low efficiency in the intestine and diaphragm. Scale bars: 250 µm (B), 200 µm (C), 400 µm (G and H).

Endothelial expression of *Pik3ca*^*H1047R*^ induces excessive lymphatic vessel sprouting and localized blood vessel dilations without sprouts in the mouse skin (Martinez-Corral et al., 2020). To explore these apparently different cellular responses of dermal lymphatic and blood ECs (LECs and BECs, respectively) to activation of PI3K signaling, we used a mouse model that allows Cre-inducible expression of *Pik3ca*^*H1047R*^ from the ubiquitously expressed *Rosa26* locus in combination with EC-specific Cre lines (Fig. 1 A). LEC-specific *Vegfr3*-*CreER*^*T2*^ (Martinez-Corral et al., 2016) and pan-endothelial *Cdh5-CreER*^*T2*^ (Wang et al., 2010) lines were complemented with a new transgenic mouse model that allows BEC-specific expression of *CreER*^*T2*^ under the control of *Flt1* (encoding VEGFR1) promoter (Fig. S1 A). Flow cytometry and immunostaining analyses confirmed that the *Vegfr1-CreER*^*T2*^ transgene drives efficient recombination of the *R26-mTmG* reporter allele and GFP expression upon tamoxifen administration specifically in BECs in the skin (Fig. S1, B–F).

To mimic the congenital *PIK3CA*-driven vascular malformations, we first induced *Pik3ca*^*H1047R*^ expression in LECs and/or BECs during embryonic development by administering 4-hydroxytamoxifen (4-OHT) to pregnant females at embryonic day (E) 10 or 11 (Fig. 1 A). As previously reported (Martinez-Corral et al., 2020), *Vegfr3*-*CreER*^*T2*^–driven activation of *Pik3ca*^*H1047R*^ expression led to hypersprouting of neuropilin-2 (NRP2)^+^ lymphatic vessels in the thoracic skin of E15 embryos, while blood vessels were not affected (Fig. 1 B). Pan-EC–specific expression of *Pik3ca*^*H1047R*^ similarly resulted in a hyperbranched lymphatic vasculature but also in multiple blood-filled lesions (Fig. 1 C) that were connected to the blood circulation as evidenced by the evacuation of blood upon application of pressure (Fig. 1 D). BEC-specific activation of *Pik3ca*^*H1047R*^ expression using the *Vegfr1*-*CreER*^*T2*^ line at E10 led to formation of blood vessel lesions that resembled those in the *Cdh5*-*CreER*^*T2*^ embryos but did not affect the lymphatic vasculature (Fig. 1 E). Whole-mount immunofluorescence staining of embryonic back skin showed localized vessel dilations that were positive for the pan-endothelial marker PECAM1 (Fig. 1 C) and the venous/capillary EC marker endomucin (EMCN; Fig. 1 E). In contrast, EMCN-negative and alpha smooth muscle actin (αSMA)–positive arteries were not affected (Fig. 1 F).

Taken together, these results demonstrate that chronic activation of p110α signaling triggers a distinct response in different dermal vessel types. Lymphatic capillaries in mutant embryos expand by sprouting, whereas blood capillaries and veins show localized vessel dilations, but arteries are not affected.

### Progressive *Pik3ca*-driven growth of postnatal dermal vasculature recapitulates vessel type–specific lesion morphology

**Figure 2.**
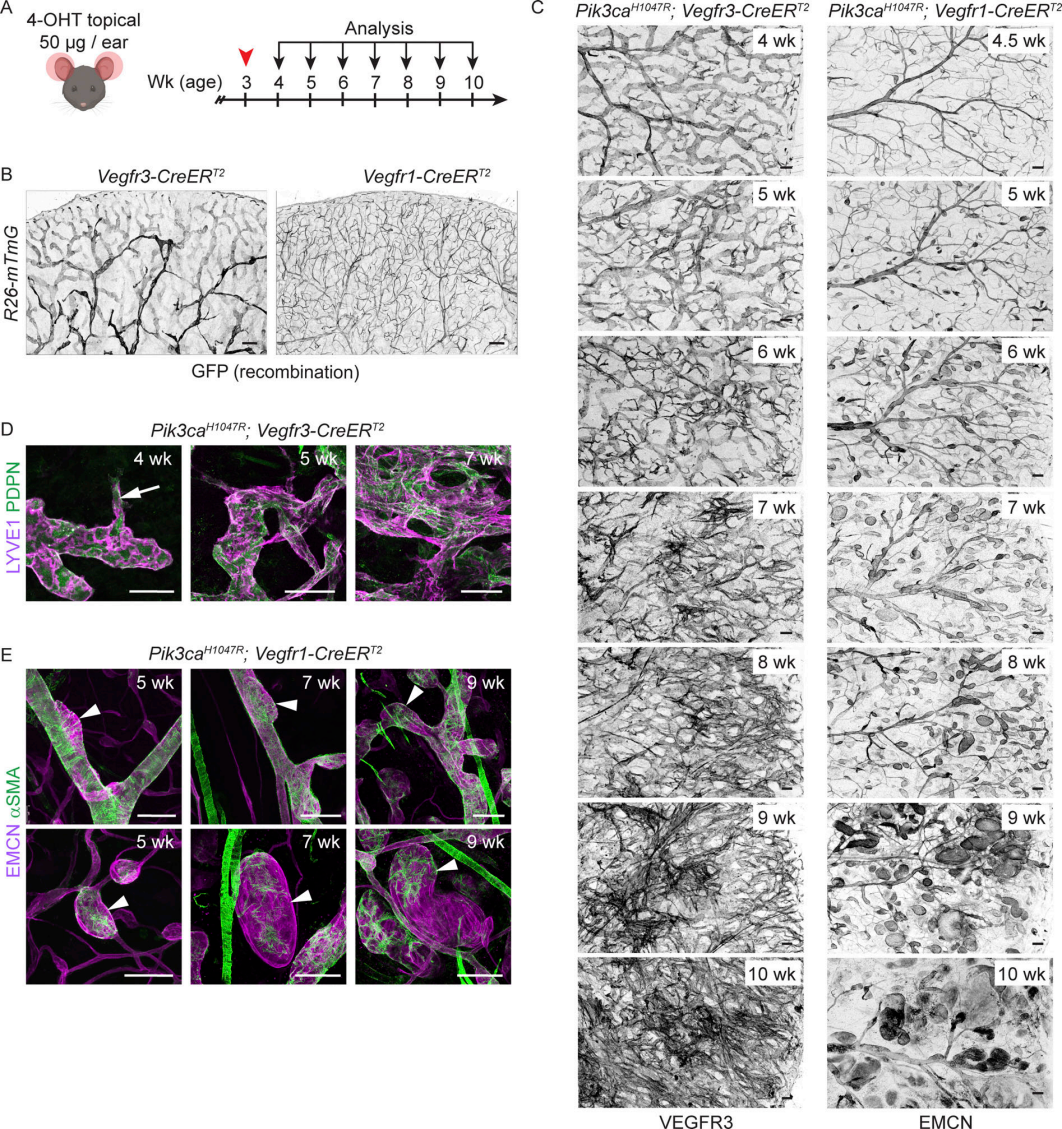
**Distinct EC-autonomous responses to oncogenic *Pik3ca* in lymphatic and blood vessels. (A)** Experimental scheme for postnatal induction of *Pik3ca*^*H1047R*^ -driven vascular overgrowth in the dermal vasculature. **(B and C)** Whole-mount staining of ears from 4-OHT–treated *Vegfr3-CreER*^*T2*^ and *Vegfr1-CreER*^*T2*^ mice in combination with the *R26-mTmG* reporter (B), or the *Pik3ca*^*H1047R*^ transgene (C), analyzed at the indicated stages after induction. Note efficient and EC type–specific recombination (GFP expression), and progressive *Pik3ca*^*H1047R*^-driven vascular overgrowth in both models. **(D and E)** Whole-mount immunofluorescence of the ear skin showing the formation of vessel sprouts (arrow) and hyperbranched lymphatic vessel network in the *Pik3ca*^*H1047R*^;*Vegfr3-CreER*^*T2*^ mice (D), as opposed to vessel dilations without sprouts (arrowheads) in veins (upper panels) and venules (lower panels) of *Pik3ca*^*H1047R*^;*Vegfr1-CreER*^*T2*^ mice (E). Note ectopic coverage by αSMA^+^ SMCs of the small lesions. Scale bar: 200 µm (B and C), 100 µm (D and E).

To allow analysis of the step-by-step development of *Pik3ca*^*H1047R*^-driven lymphatic and vascular overgrowth, we utilized postnatal mouse ear skin as a model (Martinez-Corral et al., 2020). Cre-mediated recombination was induced in 3-wk-old *Vegfr3-CreER*^*T2*^ and *Vegfr1-CreER*^*T2*^ mice by topical application of 4-OHT (Fig. 2 A). To first assess the specificity of Cre-mediated recombination, we analyzed transgenic mice carrying the *R26-mTmG* reporter allele. Efficient induction of GFP expression was observed in the ear skin vasculature with lower frequency of GFP^+^ ECs in other analyzed tissues (Fig. 2 B and Fig. S1, G and H), indicating locally restricted recombination as opposed to tissue-wide recombination observed upon systemic 4-OHT administration (Martinez-Corral et al., 2016; and data not shown). As expected, recombination was EC-subtype specific such that dermal LECs were specifically targeted in the *Vegfr3-CreER*^*T2*^ mice (Fig. 2 B and Fig. S1 G) and BECs in the *Vegfr1-CreER*^*T2*^ mice (Fig. 2 B and Fig. S1 H).

**Figure S2.**
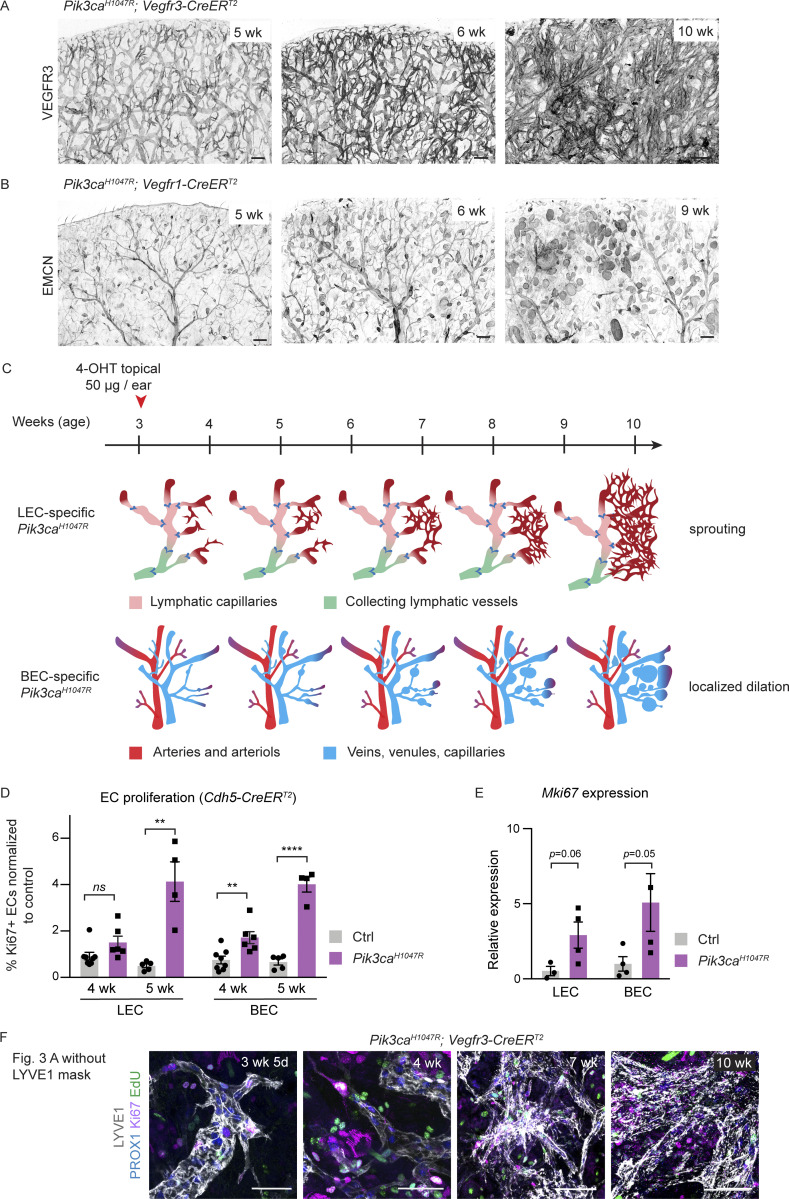
**Characterisation of a model of progressive vascular overgrowth in the *Pik3ca***^***H1047R***^***; Vegfr3-CreER***^***T2***^ **and *Pik3ca***^***H1047R***^***; Vegfr1-CreER***^***T2***^ **mice. (A and B)** Ear tile scans of the chosen time points from time-course analysis showing vascular overgrowth phenotype in *Pik3ca*^*H1047R*^;*Vegfr3-CreER*^*T2*^ (A) and *Pik3ca*^*H1047R*^;*Vegfr1-CreER*^*T2*^ (B) mice. Whole-mount immunofluorescence of LECs (VEGFR3) or (venous) BECs (EMCN) in the ear skin reveals distinct responses in lymphatic (sprouting) and blood vessels (dilation). **(C)** Schematic representation of the key features of the *Pik3ca*-driven overgrowth in lymphatic and blood vessels. **(D)** Flow cytometry analysis of proliferating dermal LECs and BECs in *Pik3ca*^*H1047R*^;*Cdh5-CreER*^*T2*^ and littermate control mice 1 or 2 wk after 4-OHT treatment (corresponding 4 or 5 wk of age respectively). Data represent mean % of Ki67^+^ ECs, normalized to the control (*n* = 4–8 mice) ± SEM. **(E)** qRT-PCR analysis of *Mki67* in dermal LECs and BECs, FACS-sorted from the ear skin of 4-OHT–treated 5-wk-old *Pik3ca*^*H1047R*^;*Cdh5-CreER*^*T2*^ and littermate control mice. Data represent mean relative expression (normalized to *Hprt*; *n* = 3–4 mice) ± SEM. Transcript levels are presented relative to control BECs and LECs. **(F)** The original unmasked images for Fig. 3 A, showing whole-mount immunofluorescence of ear skin analyzed at different stages after 4-OHT administration in *Pik3ca*^*H1047R*^;*Vegfr3-CreER*^*T2*^ mice. EdU was administered 16 h prior to analysis. P value obtained using unpaired two-tailed Student’s *t* test (D); or Mann-Whitney U test for BEC and two-tailed Student’s *t* test with Welch’s correction for LEC (E). ****, P < 0.0001; **, P < 0.01; ns, P >0.05. Scale bars: 200 μm (A and B), 50 μm (F).

Next, we analyzed the progression of the vascular phenotype upon LEC- or BEC-specific induction of *Pik3ca*^*H1047R*^ expression up to 7 wk after 4-OHT administration (i.e., at 10 wk of age; Fig. 2 A). In agreement with previous data (Martinez-Corral et al., 2020), *Vegfr3-CreER*^*T2*^–driven expression of *Pik3ca*^*H1047R*^ induced the formation of lymphatic sprouts, which progressively developed into a dense hyperbranched vessel network (Fig. 2, C and D; and Fig. S2, A and C). In contrast, and similar to the embryonic skin, ear skins of *Pik3ca*^*H1047R*^;*Vegfr1-CreER*^*T2*^ mice showed localized vessel dilations in both EMCN^+^ veins and smaller EMCN^+^ venules and capillaries (Fig. 2, C and E; and Fig. S2, B and C). The lesions progressively increased in number and size, in particular in the smaller caliber vessels (Fig. 2, C and E; and Fig. S2, B and C). EMCN^−^ αSMA^+^ arteries were not affected, but we observed abnormal coverage of the capillary/venous-derived lesions by αSMA^+^ smooth muscle cells (Fig. 2 E).

In conclusion, the analyses of the early stages of lesion formation in postnatal ear skin demonstrate different EC-autonomous responses induced by activation of p110α signaling that underlie vessel type–specific lesion morphologies. Locally limited activation of *Pik3ca*^*H1047R*^ expression induces highly reproducible lesion formation with minimal potentially life-threatening effects in the internal organs, thereby allowing an extended observation period compared to embryonic and systemic models that recapitulates human pathology.

### Oncogenic *Pik3ca* promotes sustained EC proliferation in the lymphatic vasculature

Since *Pik3ca*-driven LM and VM are somatic diseases, the initial stage of lesion formation likely involves proliferation and selective expansion of the mutant ECs. In support of this, flow cytometry analysis showed an increase in the frequency of ECs expressing the proliferation marker protein Ki67 in the *Pik3ca*^*H1047R*^;*Cdh5-CreER*^*T2*^ ear skin, which was apparent already 1 wk after 4-OHT administration and increased after 2 wk (Fig. S2 D). Quantitative RT-PCR analysis of ECs sorted by FACS from the ears of 5-wk-old *Pik3ca*^*H1047R*^;*Cdh5-CreER*^*T2*^ mice confirmed upregulation of *Mki67* (encoding Ki67) in mutant LECs and BECs compared to controls (Fig. S2 E).

**Figure 3.**
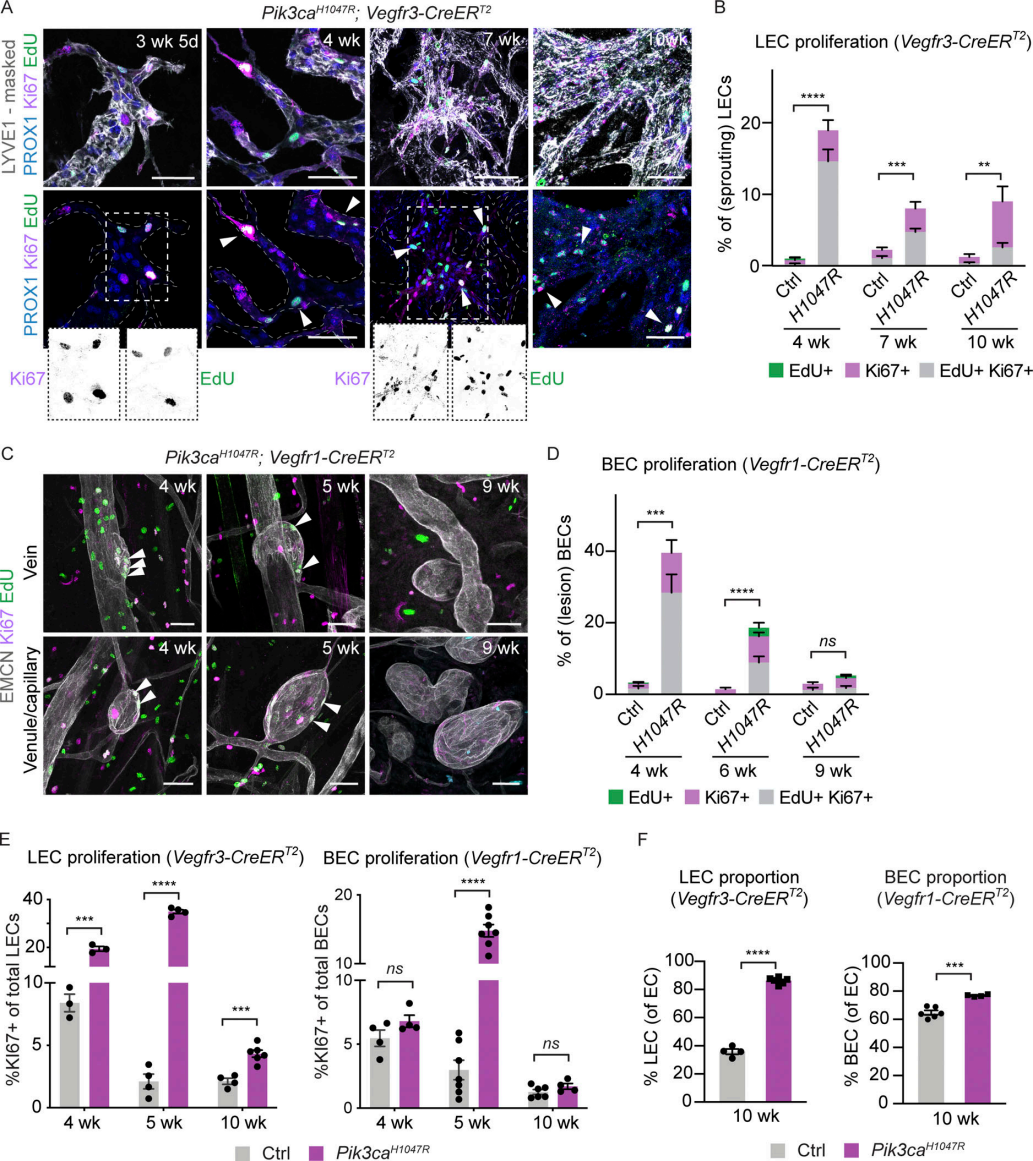
**Different proliferation dynamics of LECs and BECs during *Pik3ca*-driven vascular overgrowth. (A–D)** Whole-mount immunofluorescence of ear skin analyzed at different stages after 4-OHT administration, and quantification of S-phase cells (EdU^+^) and all cycling cells (Ki67^+^; arrowheads) in *Pik3ca*^*H1047R*^;*Vegfr3-CreER*^*T2*^ (A and B) and *Pik3ca*^*H1047R*^;*Vegfr1-CreER*^*T2*^ (C and D) mice. EdU was administered 16 h prior to analysis. LYVE1 and PROX1 were used for the identification of LECs, and EMCN for the identification of (venous) BECs. IMARIS surface mask based on LYVE1 expression was used to extract LEC-specific Ki67/EdU signals. The original unmasked images are shown in Fig. S2 F. Note initial proliferative response in both models, but sustained proliferation only in the LECs of *Vegfr3-CreER*^*T2*^ mice. **(E)** Flow cytometry analysis of proliferating dermal LECs and BECs in *Pik3ca*^*H1047R*^;*Vegfr3-CreER*^*T2*^ and *Pik3ca*^*H1047R*^;*Vegfr1-CreER*^*T2*^ mice, respectively, and their littermate controls at the indicated stages. Data represent mean % of Ki67^+^ ECs (*n* = 3–7 mice) ± SEM. **(F)** Proportion of LECs and BECs out of all ECs in the *Pik3ca*^*H1047R*^;*Vegfr3-CreER*^*T2*^ and *Pik3ca*^*H1047R*^;*Vegfr1-CreER*^*T2*^ animals analyzed in E. P value in B and D–F: two-tailed unpaired Student’s *t* test; ****, P < 0.0001; ***, P < 0.001; **, P < 0.01; ns, P > 0.05. Scale bar: 50 μm (A and C).

Next, we performed whole-mount immunofluorescence of the ear skin to localize the proliferating ECs within the abnormal vascular structures and to correlate proliferation with changes in vessel morphology. *Pik3ca*-driven vascular overgrowth was induced specifically in LECs or BECs, using the previously validated mouse models (Fig. 2 C). S-phase cells were labeled by intraperitoneal injection of EdU 16 h prior to analysis, and combined with Ki67 staining of all cycling cells. The abnormal lymphatic sprouts in the *Pik3ca*^*H1047R*^;*Vegfr3-CreER*^*T2*^ mice frequently contained proliferating LECs (Fig. 3 A and Fig. S2 F). Quantification of the frequency of PROX1^+^LYVE1^+^ LECs that were positive for EdU and/or Ki67 revealed a ∼20-fold higher level of proliferation in the mutant 1 wk after 4-OHT administration (i.e., 4 wk of age) that was sustained at approximately sixfold higher level compared to control up to at least 7 wk after induction (i.e., 10 wk of age; Fig. 3 B). A similar proliferative response was observed in BECs within developing lesions of EMCN^+^ veins and venules of *Pik3ca*^*H1047R*^;*Vegfr1-CreER*^*T2*^ mice during the first 3 wk after 4-OHT induction (Fig. 3, C and D). However, 6 wk after 4-OHT induction, i.e., at 9 wk of age, BEC proliferation rate in the lesions was reduced to that of controls (Fig. 3, C and D). Flow cytometry analysis of *Pik3ca*^*H1047R*^;*Vegfr3-CreER*^*T2*^ ear skin confirmed a sustained increase in the frequency of Ki67^+^ LECs (Fig. 3 E), and consequently a dramatic increase in the proportion of LECs of the total dermal EC population (Fig. 3 F). In contrast, the increase in Ki67^+^ BECs was observed only at the early stage of vascular lesion formation in the *Pik3ca*^*H1047R*^;*Vegfr1-CreER*^*T2*^ mice (Fig. 3 E), resulting in a small rise in total BEC numbers at 10 wk of age (Fig. 3 F).

In summary, the above data demonstrate that the initial stage of *Pik3ca*-driven vascular pathology involves increased EC proliferation both in lymphatic and blood vessels, likely through cell-autonomous mechanisms. However, in advanced lesions, the proliferation of BECs ceased whereas LEC proliferation was sustained.

### *Pik3ca*-driven LM is associated with increased myeloid cell infiltrate and cytokine levels

**Figure 4.**
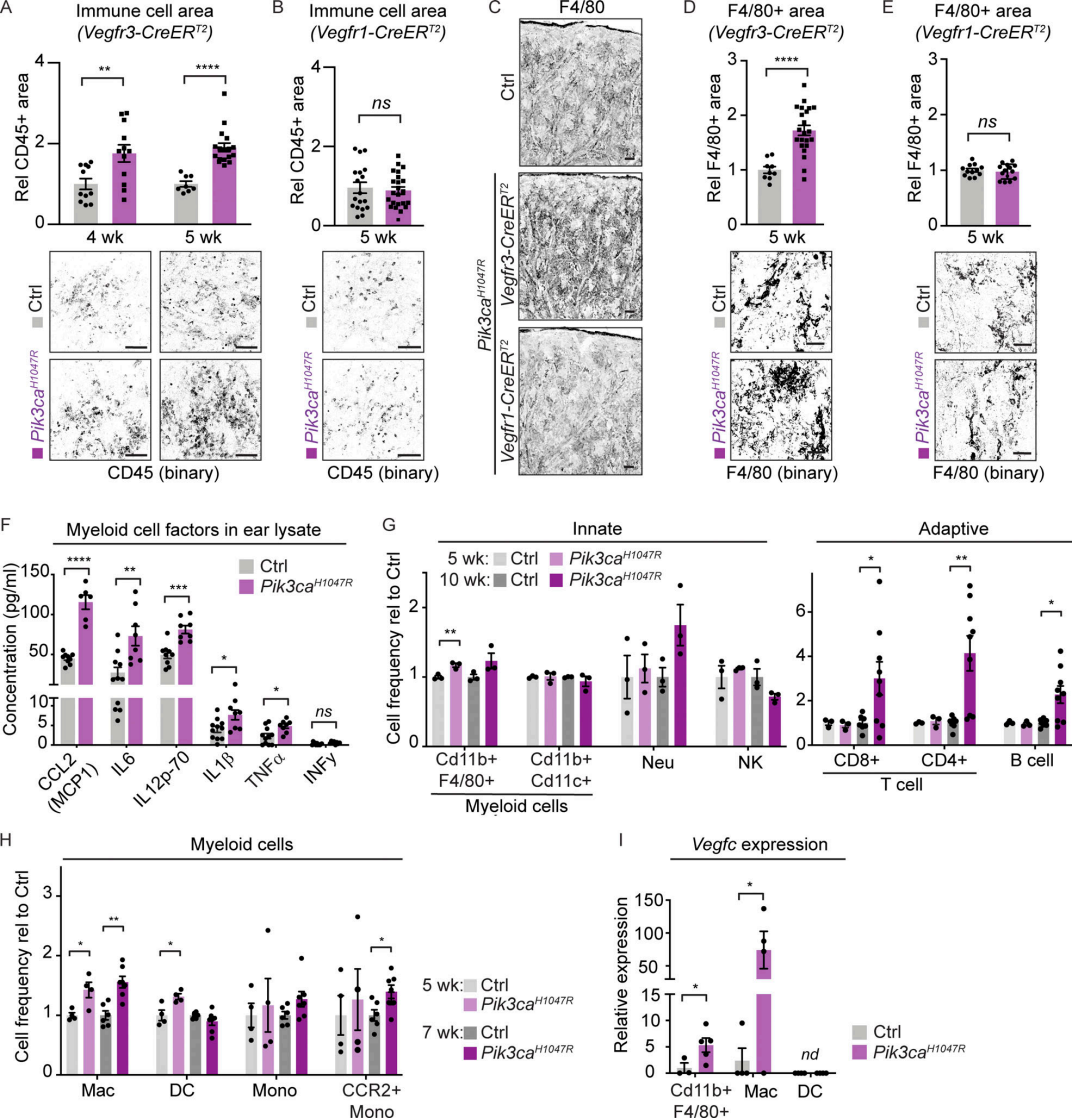
**Increased inflammatory cell infiltration and pro-inflammatory cytokine levels in the *Pik3ca*-driven LM. (A–E)** Quantification of the CD45^+^ area (A and B) and F4/80^+^ area (C–E) in the ear skin showing increase in *Pik3ca*^*H1047R*^;*Vegfr3-CreER*^*T2*^ (A, C, and D) but not in *Pik3ca*^*H1047R*^;*Vegfr1-CreER*^*T2*^ (B, C, and E) mice. Data represent mean (CD45: *n* = 4–6 images from *n* = 3–5 mice per genotype; F4/80: *n* = 3–7 images from *n* = 2–3 mice per genotype) ± SEM. Representative binary images are shown below the graphs. **(F)** Multiplex ELISA analysis of pro-inflammatory cytokines and chemokines associated with recruitment and/or activation of myeloid cells in whole-ear skin lysates from *Pik3ca*^*H1047R*^;*Vegfr3-CreER*^*T2*^ mice and littermate controls. Data represent mean protein levels relative to control (*n* = 6–11 mice) ± SEM. **(G)** Flow cytometry analysis of innate and adaptive immune cells in the ear skin of 4-OHT–treated 5- and 10-wk-old *Pik3ca*^*H1047R*^;*Vegfr3-CreER*^*T2*^ mice and littermate controls. Neu, neutrophil. Data represent cell frequency (of live cells) relative to the control (*n* = 3–9 mice) ± SEM. **(H)** Flow cytometry analysis of myeloid cells in the ear skin of 4-OHT–treated 5- and 7-wk-old *Pik3ca*^*H1047R*^;*Vegfr3-CreER*^*T2*^ mice and littermate controls. Mac, macrophage (Cd11b^+^MerTK^+^CD64^+^); DC, dendritic cell (CD11c^+^MHCII^+^); Mono, monocyte (Cd11b^+^low-SSC F4/80^+^Ly-6C^+^MHCII^+^). Data represent cell frequency (of live cells) relative to the control (*n* = 4–7 mice) ± SEM. **(I)** qRT-PCR analysis of *Vegfc* in total myeloid population, and in Mac and DC from H. Data represent mean relative expression (normalized to *Hprt*; *n* = 3–5 mice) ± SEM, presented relative to myeloid cells in control mice. nd, not detected. P value in A, B, and D–I obtained using two-tailed unpaired Student’s *t* test; for Cd11b^+^F4/80^+^ cells in I, additional Welch’s correction was used; ****, P < 0.0001; ***, P < 0.001; **, P < 0.01; *, P < 0.05; ns, P > 0.05. Scale bar: 100 μm (A–E).

To investigate the mechanisms underlying sustained proliferation of LECs in advanced LM lesions, we focused on the potential contribution of the immune infiltrate as a source of pro-lymphangiogenic factors such as VEGF-C (Harvey and Gordon, 2012; Kerjaschki, 2005). Increased abundance of immune cells, measured as CD45^+^ area, was observed in the ear skin of *Pik3ca*^*H1047R*^;*Vegfr3-CreER*^*T2*^ mice already 1 wk (i.e., 4 wk of age) after induction of vascular overgrowth (Fig. 4 A). In contrast, there was no apparent increase in CD45^+^ area around the vascular lesions in *Pik3ca*^*H1047R*^;*Vegfr1-CreER*^*T2*^ mice (Fig. 4 B). Staining for F4/80 confirmed an increased presence of myeloid cells, which constitute the majority of dermal CD45^+^ cells (Yu et al., 2016), in the *Vegfr3-CreER*^*T2*^ (Fig. 4, C and D) but not in the *Vegfr1-CreER*^*T2*^ (Fig. 4, C and E) ears 2 wk after 4-OHT induction.

**Figure S3.**
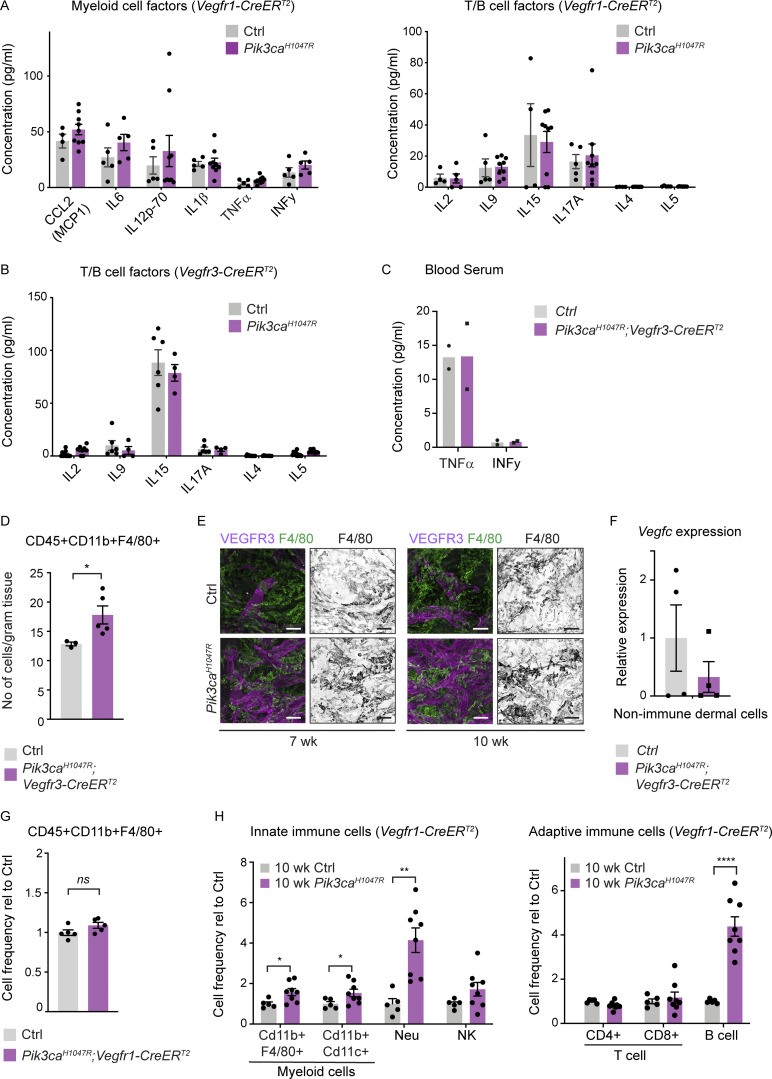
**Analysis of inflammatory cells and markers in *Pik3ca*-driven vascular lesions. (A and B)** Multiplex ELISA analysis of pro-inflammatory cytokines and chemokines associated with recruitment and/or activation of myeloid cells or T cells and B cells in whole-ear skin lysates from *Pik3ca*^*H1047R*^;*Vegfr1-CreER*^*T2*^ (A) and *Pik3ca*^*H1047R*^;*Vegfr3-CreER*^*T2*^ (B) mice, and respective littermate controls. **(C)** Similar analysis of TNFα and INFγ in blood serum of *Pik3ca*^*H1047R*^;*Vegfr3-CreER*^*T2*^ mice. Data in A–C represent mean protein levels (*n* = 3–9 mice [A and B]) ± SEM, or *n* = 2 mice (C). **(D)** Flow cytometry analysis of the number of CD45^+^CD11b^+^F4/80^+^ macrophages in the ear skin of 4-OHT–treated 5-wk-old *Pik3ca*^*H1047R*^;*Vegfr3-CreER*^*T2*^ (*n* = 5) and control (*n* = 3) mice. Data represent mean cell number per gram tissue ± SEM. P value, Mann–Whitney U test. **(E)** Whole-mount immunofluorescence of ears from a control (Ctrl) mouse, and 7- or 10-wk-old 4-OHT–treated *Pik3ca*^*H1047R*^;*Vegfr3-CreER*^*T2*^ mice for myeloid marker F4/80. **(F)** qRT-PCR analysis of *Vegfc* in live non-immune dermal cell population (gated out all myeloid cells, neutrophils, NK cells, B cells, and T cells using panels shown in Fig. S5 D). Data represent mean relative expression (normalized to *Hprt*; *n* = 4 mice) ± SEM, presented relative to control mice. **(G)** Flow cytometry analysis of the frequency of CD45^+^CD11b^+^F4/80^+^ myeloid cells in the ear skin of 4-OHT–treated 5-wk-old *Pik3ca*^*H1047R*^;*Vegfr1-CreER*^*T2*^ (*n* = 6) and control (*n* = 5) mice. Data represent relative cell frequency (of live cells) relative to the control ± SEM. P value obtained using two-tailed unpaired Student’s *t* test. **(H)** Flow cytometry analysis of innate (left) and adaptive (right) immune cells in the ear skin of 4-OHT–treated 10-wk-old *Pik3ca*^*H1047R*^*; Vegfr1-CreER*^*T2*^ mice and littermate controls. Neu, neutrophil. Data represent relative cell frequency (of live cells) relative to the control (*n* = 5–8 mice for innate panel, *n* = 7–9 mice for adaptive panel) ± SEM. P value (in D and H) obtained using two-tailed unpaired Student’s *t* test. ****, P < 0.0001; **, P < 0.01; *, P < 0.05; ns, P > 0.05. Scale bar: 50 μm (E).

To assess the inflammatory status of the ears, we performed multiplex ELISA that allows simultaneous measurement of multiple pro-inflammatory cytokines and chemokines. The levels of pro-inflammatory proteins associated with the recruitment and/or activation of antigen-presenting myeloid cells, including CCL2 (also known as monocyte chemoattractant protein MCP1), IL1β, IL6, and IL12, were significantly increased in ear skin lysates of 5-wk-old *Pik3ca*^*H1047R*^;*Vegfr3-CreER*^*T2*^ mice (Fig. 4 F), but not of *Pik3ca*^*H1047R*^;*Vegfr1-CreER*^*T2*^ mice (Fig. S3 A). Proteins associated with the recruitment and/or activation of B cells (IL4, IL5) or T cells (IL2, IL9, IL15, IL17A) were unaltered in both models (Fig. S3, A and B). The major pro-inflammatory cytokines TNFα and INFγ were also unaltered in the blood sera of *Pik3ca*^*H1047R*^;*Vegfr3-CreER*^*T2*^ mice (Fig. S3 C), thereby excluding systemic inflammation. TNFα levels were increased in the mutant in comparison with control ear skin lysate (Fig. 4 F), but the low concentration likely reflects low-grade local chronic inflammation (Li et al., 2009).

### Recruitment of VEGF-C–producing macrophages in *Pik3ca*-driven LM

Further analysis of innate and adaptive immune cells by flow cytometry showed increase in the frequency (Fig. 4 G and Data S1) and number (Fig. S3 D) of CD45^+^CD11b^+^F4/80^+^ myeloid cells in the ears of *Pik3ca*^*H1047R*^;*Vegfr3-CreER*^*T2*^ mice compared to controls. Additional myeloid markers revealed selective increase in macrophages (Cd11b^+^MerTK^+^CD64^+^) at two time points of disease progression at 5 and 7 wk of age (Fig. 4 H). The frequency of dendritic cells (MerTK^−^CD64^−^CD11c^+^MHCII^+^) was modestly increased only at 5 wk, while the total monocyte (Cd11b^+^low-SSC F4/80^+^Ly-6C^+^MHCII^+^) abundance was not significantly altered at either time point (Fig. 4 H). Interestingly, however, the frequency of monocytes expressing the CCL2 ligand CCR2 that represented 20–30% of the total monocyte population was increased in mutant in comparison with control ears at 7 wk of age (Fig. 4 H). Advanced lesions, analyzed by FACS at 10 wk of age (7 wk after 4-OHT administration) additionally showed an increased frequency of T cells (CD45^+^CD3^+^CD4/CD8^+^) and B cells (CD45^+^CD3^−^B220^+^NK1.1^−^), while neutrophils (CD45^+^CD11b^+^Ly6G^+^) or natural killer (NK) cells (CD45^+^CD3^−^NK1.1^+^) did not show significant alterations (Fig. 4 G). Immunofluorescence staining confirmed an increased abundance of F4/80^+^ myeloid cells in the *Pik3ca*^*H1047R*^;*Vegfr3-CreER*^*T2*^ ears until the analysis at 10 wk of age (Fig. S3 E).

Selective myeloid cell recruitment during the first weeks of *Pik3ca*-driven vessel growth was only observed in LM, since CD45^+^CD11b^+^F4/80^+^ myeloid cells were not significantly increased in the ears of *Pik3ca*^*H1047R*^;*Vegfr1-CreER*^*T2*^ mice compared to controls at 5 wk of age (Fig. S3 G). Advanced venous lesions at 10 wk of age instead showed an increased frequency of several immune populations including myeloid cells, but also, and different from LM, neutrophils, and B cells (Fig. S3 H). The delayed immune response is likely secondary to disruption of vessel integrity and leakage in this model.

To assess the pro-lymphangiogenic nature of the myeloid infiltrate in *Pik3ca*^*H1047R*^;*Vegfr3-CreER*^*T2*^ ears, we assessed *Vegfc* transcript levels. qRT-PCR analysis of FACS-sorted CD45^+^CD11b^+^F4/80^+^ myeloid cells showed higher *Vegfc* levels in mutant in comparison with control mice (Fig. 4 I). Notably, macrophages in mutant ears showed a strong increase in *Vegfc* expression, while the levels were low and not altered in dendritic cells (Fig. 4 I) as well as in total CD45^−^ non-immune dermal cells (Fig. S3 F).

Collectively, the above data demonstrate that infiltration of macrophages, as well as the upregulation of pro-inflammatory cytokines and chemokines promoting their recruitment, are selective features of and account for increased production of *Vegfc* in *Pik3ca*^*H1047R*^-driven LM.

### Single-cell transcriptomics identifies a molecularly distinct dermal capillary LEC subtype with putative immune functions

**Figure 5.**
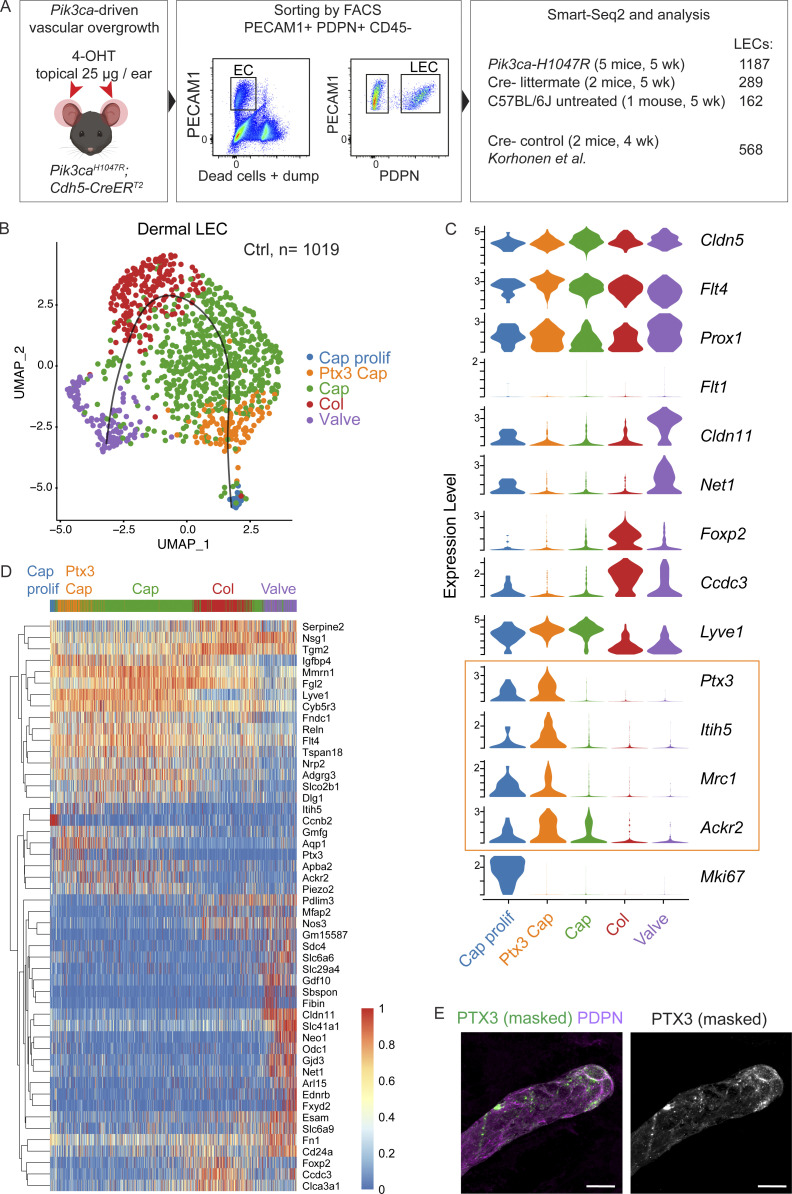
**Definition of dermal LEC subtypes by single cell transcriptomics. (A)** Schematic overview of dermal LEC isolation for scRNA-seq. **(B)** The five dermal LEC clusters from control ear skin visualized in a UMAP landscape, labeled by cluster assignment. Black line depicts trajectory calculated from UMAP embedding (unsupervised Slingshot algorithm). **(C)** Violin plots showing the expression of selected pan-EC, LEC, and BEC markers, as well as LEC subtype marker genes in the five clusters. Note a new LEC subtype (immune-interacting LECs, iLECs; yellow box) defined by the expression of *Ptx3*, *Itih5*, and *Mrc1* that are also expressed in *Ptx3*-LECs in the lymph node. **(D)** Heatmap showing the expression of zonation markers across the five LEC clusters. Cells were ordered by the trajectory. Color indicates read counts in log-scale. **(E)** Whole-mount immunofluorescence of non-permeabilized ear skin showing extracellular PTX3 staining predominantly at capillary terminals. IMARIS surface mask based on podoplanin (PDPN) expression was used to extract LEC-specific PTX3 signal. The original unmasked image is shown in Fig. S4 E. Scale bar: 20 μm (H).

The pro-inflammatory molecules specifically upregulated in the *Pik3ca*^*H1047R*^;*Vegfr3-CreER*^*T2*^ skin are expressed in a variety of cell types, including the infiltrating myeloid cell themselves (Farnsworth et al., 2019). To determine the contribution of LEC-autonomous PI3K signaling in promoting a pro-inflammatory environment, we applied single-cell RNA sequencing (scRNA-seq). LECs were isolated by flow cytometry from the ear skin of *Pik3ca*^*H1047R*^;*Cdh5-CreER*^*T2*^ (*n* = 5) and Cre^−^ littermate (*n* = 2) mice 2 wk after 4-OHT treatment, and subjected to scRNA-seq using Smart-Seq2 (Picelli et al., 2013; Fig. 5 A). Additional controls included a separately bred untreated wild-type (C57BL/6J) mouse to control for possible effects of the treatment in littermate controls, and 2–4-wk-old mice from a previous study (Korhonen et al., 2022).

**Figure S4.**
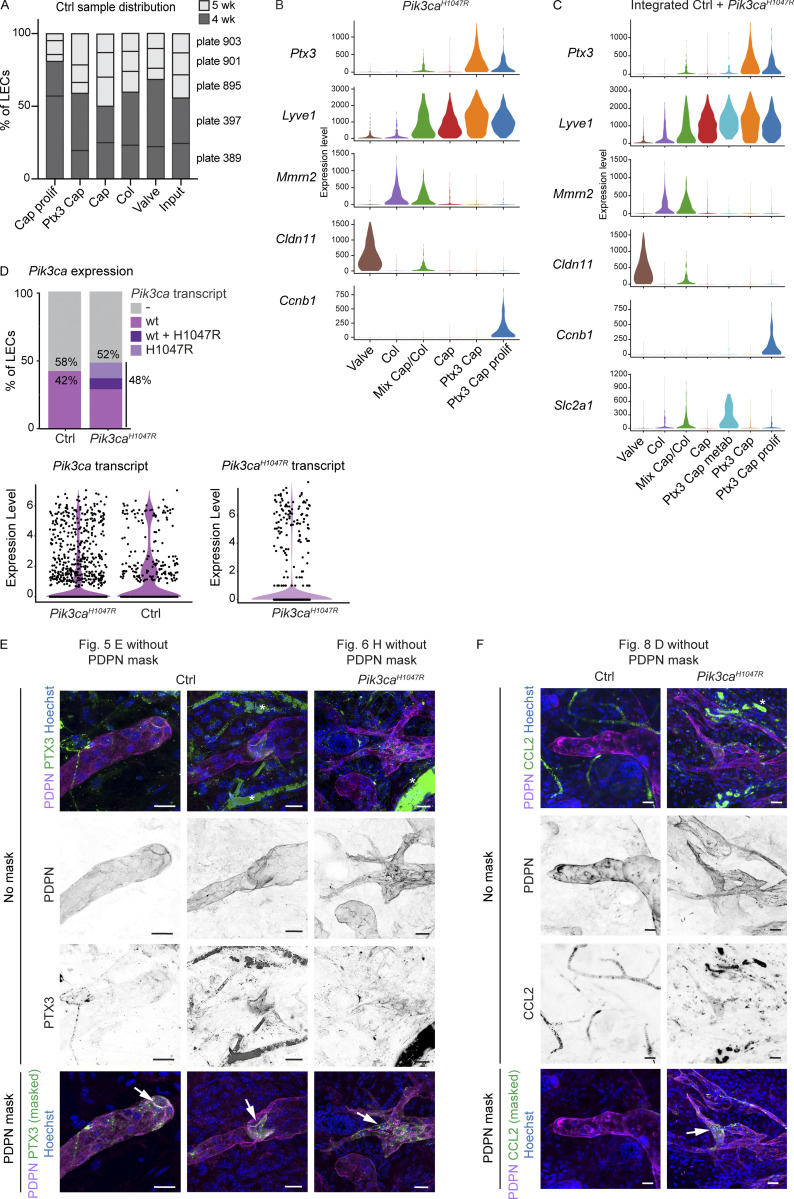
**Characterization of cell clusters in scRNA-seq datasets of *Pik3ca***^***H1047R***^ **mutant and control LECs. (A)** Contribution of cells from different control mice (plates) in the five clusters of normal LECs. Input levels of each plate are shown, and color code indicates mouse age. **(B and C)** Violin plots showing the expression of selected LEC and LEC subtype marker genes in LEC clusters from the *Pik3ca*^*H1047R*^ mutant dataset (from Fig. 6 A; B) and integrated dataset containing LECs from control and *Pik3ca*^*H1047R*^;*Cdh5-CreER*^*T2*^ mice (from Fig. 6 D; C). **(D)** Proportion of LECs from control and *Pik3ca*^*H1047R*^;*Cdh5-CreER*^*T2*^ mice expressing the endogenous mouse *Pik3ca* transcript (control and mutant mice), and/or the transgenic *Pik3ca*^*H1047R*^ transcript (mutant mice only). Violin plots below show the expression levels. Note that the majority of LECs lack the *Pik3ca* transcript. **(E and F)** Whole-mount immunofluorescence of control and *Pik3ca*^*H1047R*^;*Vegfr3-CreER*^*T2*^ ear skin showing high expression of PTX3 (E) and CCL2 (F) in the capillary terminals (Ctrl) and abnormal lymphatic sprouts (mutant; arrows). PTX3 staining is also observed in lymphatic valves (arrow). IMARIS surface mask based on PDPN expression was used to extract LEC-specific signals (images below, shown in the main figures). The original images including single channels for PDPN and PTX3 (E) or CCL2 (F) are shown (no mask). Unspecific signal from red blood cells is indicated by asterisks. Scale bars: 20 µm (E and F).

To define the normal transcriptome of dermal LECs, we analyzed 1,019 single-cell transcriptomes from control mice that passed the quality controls. The cells distributed into five clusters after applying the canonical correlation analysis method for batch correction and Seurat graph–based clustering approach (Stuart et al., 2019), and visualization using the Uniform Manifold Approximation and Projection (UMAP; McInnes et al., 2020; Fig. 5 B). As expected, all clusters were characterized by high expression of pan-endothelial (*Cldn5*) and LEC-specific marker genes (*Flt4*, *Prox1*), and lack of BEC marker expression (*Flt1*; Fig. 5 C). Based on the expression of known LEC subtype markers, the clusters were annotated as valve LECs (*Cldn11*^*+*^; Takeda et al., 2019), collecting vessel LECs (*Foxp2*^*+*^; Hernández Vásquez et al., 2021), and capillary LECs (*Lyve1*^*high*^; Fig. 5 C). In addition, we observed a previously unknown population of LECs defined by high expression of *Ptx3*, which encodes the humoral pattern recognition molecule Pentraxin 3 (Doni et al., 2016). *Ptx3*^*high*^ LECs included two clusters of non-proliferating and proliferating *Lyve1*^*high*^ capillary LECs, the latter recognized by their high expression of cell-cycle genes (e.g., *Mki67*; Fig. 5 C). Interestingly, *Ptx3* was recently shown to define a subpopulation of lymph node LECs, characterized by high expression of genes involved in the regulation of lymphangiogenesis and immune response (Xiang et al., 2020). *Ptx3*^*high*^ dermal LECs shared a set of their marker genes and were enriched in transcripts encoding regulators of innate and adaptive immune responses including *Ptx3* itself, as well as phagocytic pathogen (*Mrc1*) and chemokine (*Ackr2*) receptors, and regulators of T cell activation (*Cd276*, *Cd200*; Fig. 5 C and Data S2). Additional cluster markers are shown (Fig. 5 D) and listed (Data S2), and the data are available for browsing at https://makinenlab.shinyapps.io/DermaLymphaticEndothelialCells/. The proportion of cells that contributed to each cluster by the five control samples was proportional to the input, except for the proliferative cluster that was composed mainly of LECs isolated from the younger 4-wk-old mice (Fig. S4 A).

Ordering of cells based on similarities in their expression patterns generated a linear trajectory across the clusters with *Ptx3*^*high*^ LECs at the end of the trajectory (Fig. 5, B and D; and Data S3). The observed phenotypic progression, termed “zonation” (Fig. 5 D), mirrors anatomical positioning of so-called pre-collectors that share molecular and functional features of lymphatic capillaries and collecting vessels (Petrova and Koh, 2020). Whole-mount immunofluorescence of non-permeabilized ear skin of wild-type mouse revealed high cell surface PTX3 at blunt-ended terminals of lymphatic capillaries (Fig. 5 E and Fig. S4 E), thereby indicating a distinct anatomical location of PTX3^+^ LECs in normal vasculature. A subset of valves of pre-collecting vessels was also PTX3^+^ (Fig. S4 E).

In summary, scRNA-seq identifies dermal LEC hierarchy that recapitulates lymphatic vascular architecture and defines a previously unknown molecularly distinct *Ptx3*^*high*^ population within dermal capillary terminals as a putative immune-interacting LEC subtype—termed here as iLEC.

### *Pik3ca*^*H1047R*^ drives iLEC expansion

**Figure 6.**
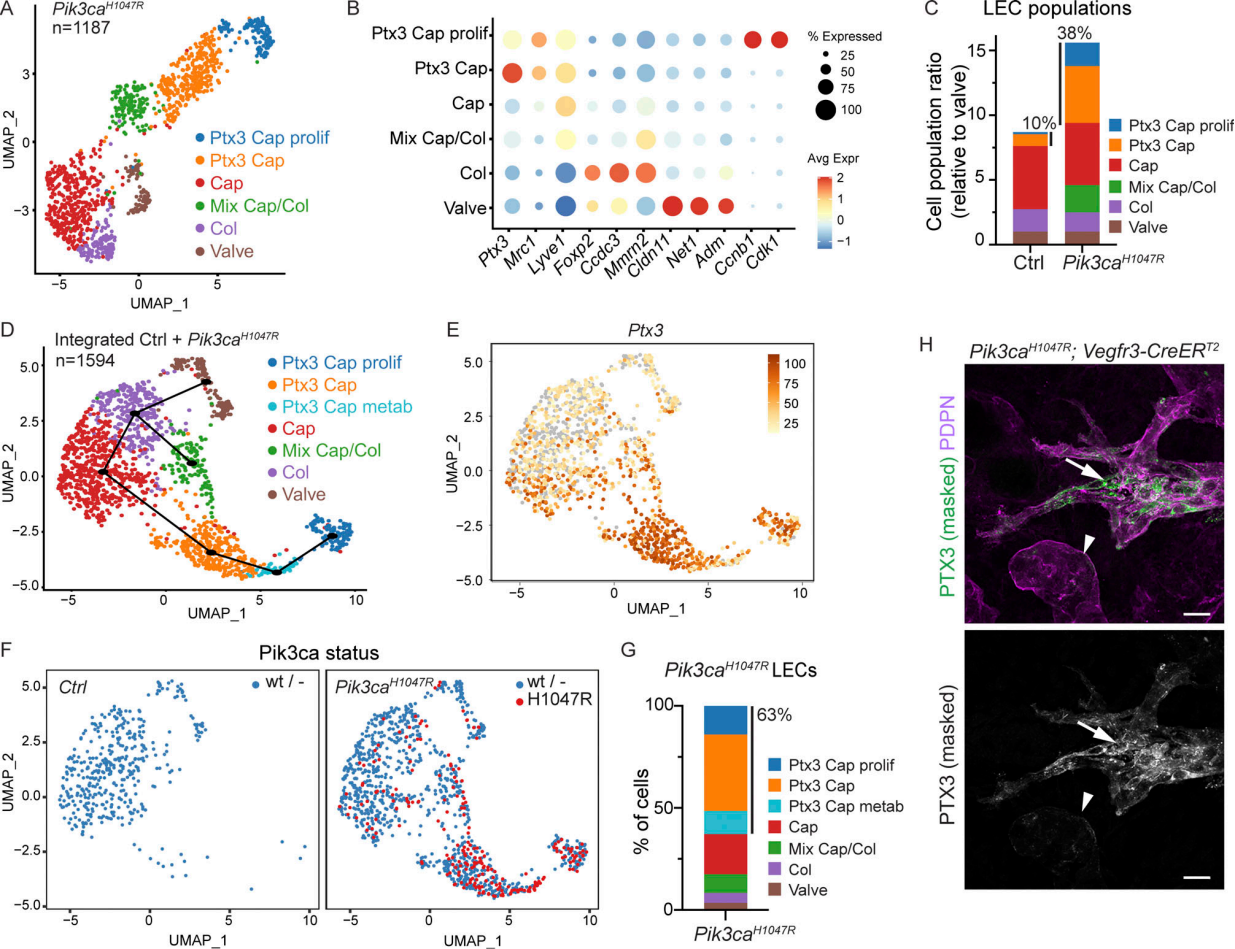
**Expansion of *Ptx3***^***+***^ **iLEC population in *Pik3ca*-driven LM. (A)** The six dermal LEC clusters from *Pik3ca*^*H1047R*^;*Cdh5-CreER*^*T2*^ ear skin visualized in a UMAP landscape. **(B)** Dot plot of LEC subtype markers for the six LEC clusters from A. Dot size illustrates percentage of cells presenting transcript sequence counts and color illustrates the average expression level (log_2_ fold change) within a cluster. **(C)** Subtype composition of LEC populations in the control and *Pik3ca*^*H1047R*^ mutant skin. Population sizes are shown relative to valve LECs (= 1). The percentage of *Ptx3*^*+*^ capillary LEC population of the total LEC population is indicated. **(D)** The seven dermal LEC clusters from the dataset integrating data from control and *Pik3ca*^*H1047R*^;*Cdh5-CreER*^*T2*^ ear skin visualized in a UMAP landscape. Black line depicts trajectories calculated from UMAP embedding of the combined dataset (unsupervised Slingshot algorithm), resulting in two branches. **(E)** *Ptx3* expression visualized in a UMAP. **(F)** Cell distribution based on genotype and *Pik3ca* transcript status visualized in a UMAP, as indicated. *Ptx3*^*+*^ capillary LEC clusters from *Pik3ca*^*H1047R*^ mutant mice are enriched with cells expressing the mutant transcript (red). **(G)** Distribution of LECs expressing the mutant *Pik3ca*^*H1047R*^ transcript showing the majority (63%) within the three Ptx3^high^ clusters. **(H)** Whole-mount immunofluorescence of *Pik3ca*^*H1047R*^;*Vegfr3-CreER*^*T2*^ ear skin showing high expression of PTX3 in the abnormal lymphatic sprouts (arrows) compared to morphologically normal lymphatic capillaries (arrowheads). IMARIS surface mask based on PDPN expression was used to extract LEC-specific signals. The original unmasked images are shown in Fig. S4 E. Scale bars: 20 μm (H).

Next, we performed a similar analysis of LECs isolated from the *Pik3ca*^*H1047R*^;*Cdh5-CreER*^*T2*^ mice. We obtained, in total, 1,187 quality-controlled single-cell transcriptomes that distributed into six clusters (Fig. 6 A). Based on the expression of the LEC subtype markers identified in the control skin dataset (Data S2), we defined clusters of valve, collecting vessel and capillary LECs. We also observed a large population of *Ptx3*^*high*^ capillary LECs that included two clusters of non-proliferating and proliferating LECs (Fig. 6, A and B; and Fig. S4 B). One cluster was characterized by an intermediate identity with expression of both capillary (e.g., *Lyve1*) and collecting vessel (e.g., *Mmrn2*) genes (Fig. 6, A and B; and Fig. S4 B). Enriched genes for each cluster are listed in Data S4. Cluster level analysis thus suggested active expansion of the *Ptx3*^*high*^ iLEC population in *Pik3ca*^*H1047R*^ mutant mice, which was also evident in the subtype composition of LEC populations in the mutant in comparison with control skin (Fig. 6 C).

For the identification of potential pathological cell populations, we integrated the LEC single-cell transcriptomes from 5-wk-old control and *Pik3ca*^*H1047R*^ mutant mice (after removal of contaminants in total 1,594 cells) using Harmony and identified seven LEC clusters (Fig. 6 D). Marker expression defined six clusters corresponding to the same identities than those in the *Pik3ca*^*H1047R*^ mutant mice, including non-proliferating and proliferating *Ptx3*^*high*^ capillary LECs (Fig. 6, D and E; Fig. S4 C; and Data S5), but also an additional *Ptx3*^*high*^ cluster characterized by high expression of metabolic genes (Fig. S4 C and Data S5). Trajectory analysis based on gene expression data suggested linear phenotypic progression between the non-proliferative and proliferative *Ptx3*^*high*^ clusters (Fig. 6 D). Assessment of the relative contribution of cells originating from the different genotypes of mice revealed that the *Ptx3* capillary LEC clusters, as well as the cluster of mixed identity, were almost exclusively composed of LECs isolated from the *Pik3ca*^*H1047R*^ mutant mice (Fig. 6, F and G). *Ptx3*^*high*^ capillary LEC clusters further showed enrichment of cells expressing the transgene-encoded *Pik3ca*^*H1047R*^ transcript (Fig. 6, F and G; and Fig. S4 D), which was expressed at a similar level compared to the endogenous mouse *Pik3ca* transcript (Fig. S4 D). Whole-mount immunofluorescence confirmed increased expression of PTX3 (Fig. 6 H and Fig. S4 E) in the abnormal lymphatic vessel sprouts in *Pik3ca*^*H1047R*^;*Vegfr3-CreER*^*T2*^ mice, further supporting the selective expansion of the *Ptx3* capillary iLEC population in the mutant skin.

### Increased PTX3 expression in human LM

**Figure 7.**
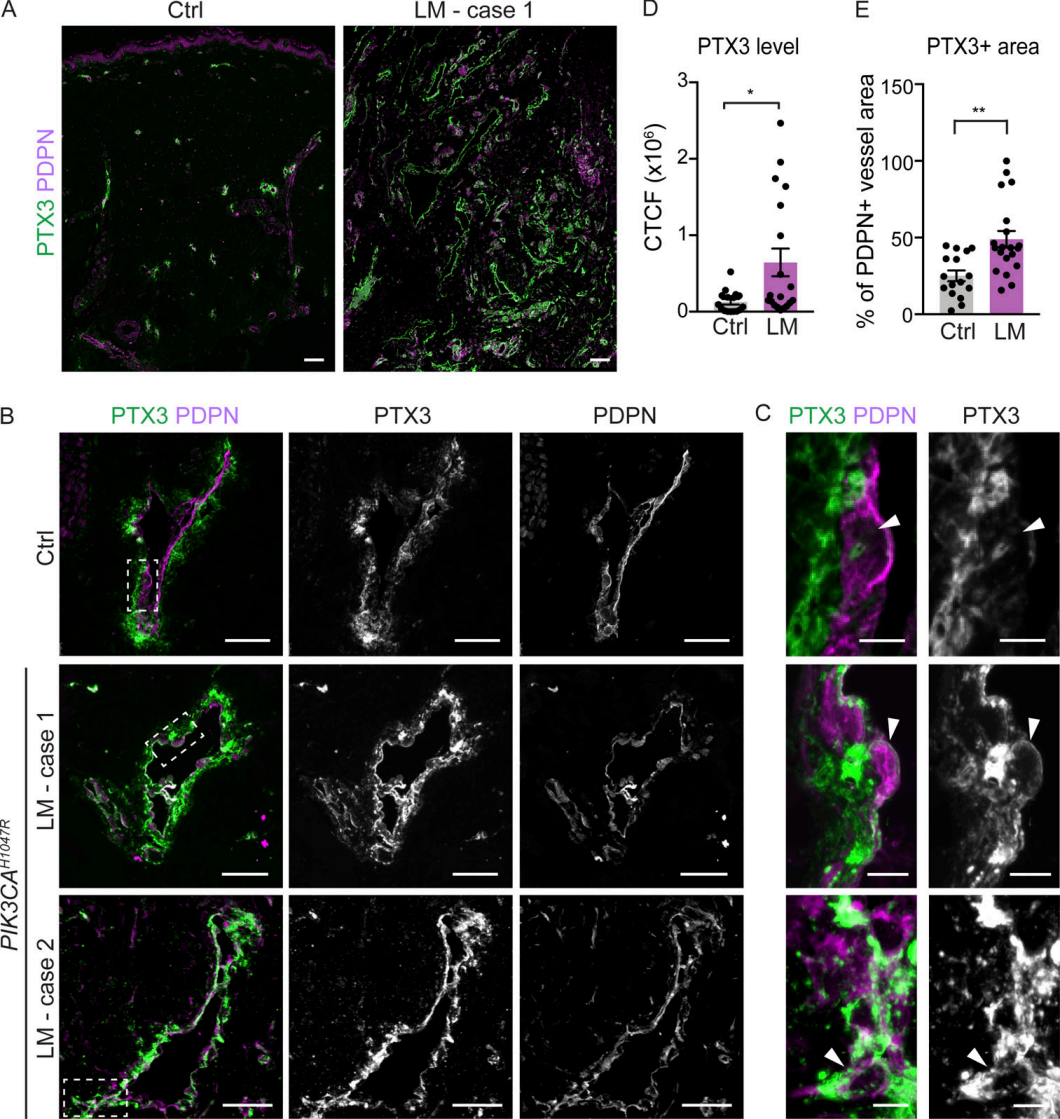
**Increased PTX3 deposition and expression in human *PIK3CA***^***H1047R***^ **-driven LM. (A–C)** Immunofluorescence staining of PTX3 in paraffin sections of normal skin and LMs with a *PIK3CA*^*H1047R*^ mutation. Boxed areas in B are magnified in C. Note deposition of PTX3 around PDPN^+^ lymphatic vessels in control tissue but low expression in LECs, while LECs from LM tissue show PTX3 immunoreactivity (arrowheads in C). **(D and E)** Quantification of PTX3 immunoreactivity in PDPN^+^ lymphatic vessels showing higher intensity, measured as corrected total cell fluorescence (CTCF; D), and larger PTX3^+^ vessel area (E) in LECs from LM in comparison with control tissue. P value in D and E: two-tailed unpaired Student’s *t* test; **, P < 0.01; *, P < 0.05. Scale bars: 100 μm (A), 50 μm (B), 5 μm (C).

To explore potential clinical relevance of the findings, we analyzed PTX3 expression in normal human skin and in LMs. Clinical features of three LM patients with a *PIK3CA*^*H1047R*^ mutation selected for the study are summarized in Table S1. Immunofluorescence staining of paraffin sections of normal skin showed deposition of PTX3 around PDPN^+^ lymphatic vessels in control tissue but low levels in LECs themselves (Fig. 7, A–C). In contrast, LECs within LM lesions showed strong immunoreactivity (Fig. 7, A–C). PTX3 immunostaining intensity, measured as corrected total cell fluorescence, was fivefold higher in LECs from LM in comparison with control tissue (Fig. 7 D), and covered a twofold larger area of PDPN^+^ lymphatic vessels (Fig. 7 E).

Taken together, the expansion and active proliferation of the *Ptx3*^*high*^ capillary iLECs in the mouse model of *Pik3ca*^*H1047R*^-driven LM, and high lymphatic endothelial expression and deposition of PTX3 in human LM suggest PTX3^high^ LECs as pathogenic cells in these vascular lesions.

### *Pik3ca*^*H1047R*^ induces a pro-inflammatory transcriptome in iLECs

**Figure 8.**
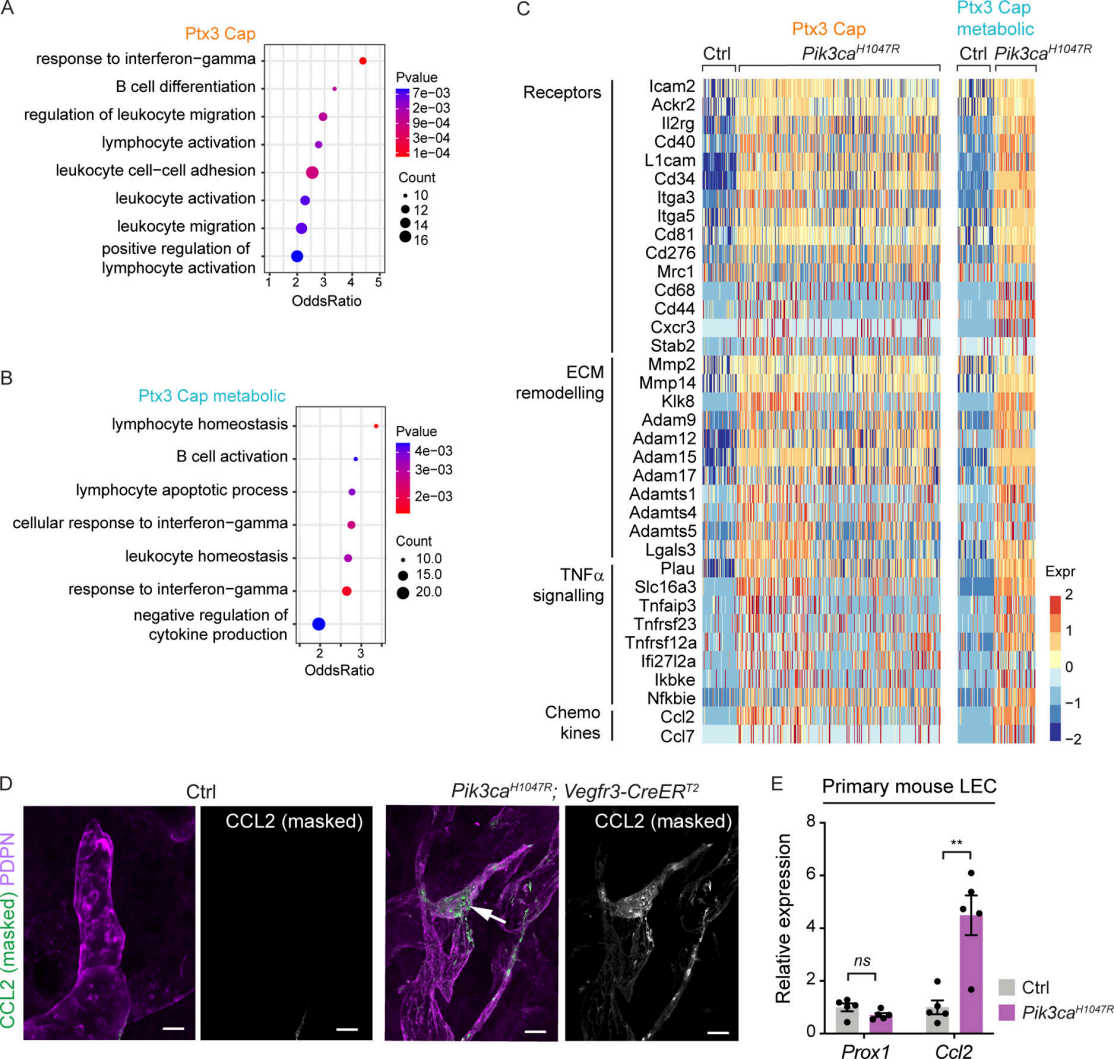
**Pro-inflammatory transcriptome of *Pik3ca***^***H1047R***^ **-expressing iLECs. (A and B)** GO analysis of genes enriched in the clusters of non-proliferative *Ptx3*^*+*^ capillary LECs from *Pik3ca*^*H1047R*^ in comparison with *Ptx3*^*+*^ capillary LECs in control mice. Selected terms for enriched immune-related biological process are shown. Dot size illustrates count number and color illustrates adjusted P value. **(C)** Heatmap showing the expression of upregulated immune-related genes in *Ptx3*^*+*^ capillary LECs from *Pik3ca*^*H1047R*^ mutant in comparison with control mice. Color illustrates the expression level (log_2_ fold change). **(D)** Whole-mount immunofluorescence of *Pik3ca*^*H1047R*^;*Vegfr3-CreER*^*T2*^ ear skin showing lymphatic endothelial expression of CCL2/MCP1 in the mutant. **(E)** qRT-PCR analysis of primary dermal LECs isolated from *Pik3ca*^*H1047R*^;*Vegfr3-CreER*^*T2*^ ears, showing increased *Ccl2* expression in *Pik3ca*^*H1047R*^-expressing (with 4-OHT) in comparison with control (without 4-OHT) LECs. *Prox1* levels were unchanged. Data represent mean relative expression (normalized to *Hprt*; *n* = 5 samples) ± SEM, presented relative to control cells. P value obtained using hypergeometric test (A and B) and two-tailed unpaired Student’s *t* test (E). **, P < 0.01; ns, P > 0.05. Scale bars: 20 μm (D).

To identify LEC-autonomous *Pik3ca*-driven transcriptional changes, we next focused on the pathological *Ptx3* capillary LEC clusters representing iLECs. To avoid the confounding effect of the cell cycle (Chen and Zhou, 2017), we determined differentially expressed genes (DEG) between the non-proliferative *Ptx3* clusters in mutant mice in comparison with *Ptx3* capillary LECs from control mice (Data S6). Gene Ontology (GO) analysis of DEGs revealed enrichment of biological processes related to metabolic processes, cell-cycle transition, cell migration, and cell-matrix adhesion in the mutant clusters (Datas S7 and S8), consistent with the established role of the PI3K pathway (Graupera and Potente, 2013) and previously reported migratory phenotype of *Pik3ca*^*H1047R*^-expressing LECs in vitro and in vivo (Martinez-Corral et al., 2020). Both mutant clusters also showed enrichment of processes and genes related to immune regulation (Fig. 8, A and B; and Data S7 and S8). The latter include upregulation of genes encoding pro-inflammatory cytokines (*Ccl2*, *Ccl7*), (scavenger) receptors (*Ackr2*, *L1cam*), as well as extracellular matrix proteins (*Lgals3*) and proteinases (*Adam17*, *Adam8*, *Mmp14*, *Mmp2*) implicated in inflammatory processes (Fig. 8 C and Data S6). Conversely, downregulated biological processes include negative regulation of inflammatory processes. Lymphatic endothelial expression of the key monocyte/macrophage chemokine CCL2/MCP1 in *Pik3ca*^*H1047R*^;*Vegfr3-CreER*^*T2*^ mice was confirmed by whole-mount immunofluorescence, while no staining was detected in the control skin (Fig. 8 D and Fig. S4 F).

To assess if oncogenic PI3K directly regulates *Ccl2* expression in LECs, in the absence of immune cells, we isolated primary dermal LECs from *Pik3ca*^*H1047R*^;*Vegfr3-CreER*^*T2*^ mice and analyzed transcript levels by qRT-PCR after induction of Cre recombination by supplementation of 4-OHT to the culture medium. We observed a significant upregulation of *Ccl2* transcript after induction of *Pik3ca*^*H1047R*^ expression, while the levels of the general LEC marker *Prox1* were not altered (Fig. 8 E).

Taken together, single-cell transcriptomics revealed that *Pik3ca*^*H1047R*^ promotes a pro-inflammatory transcriptome in LECs.

### Macrophage depletion and anti-inflammatory therapy limit LM growth

**Figure 9.**
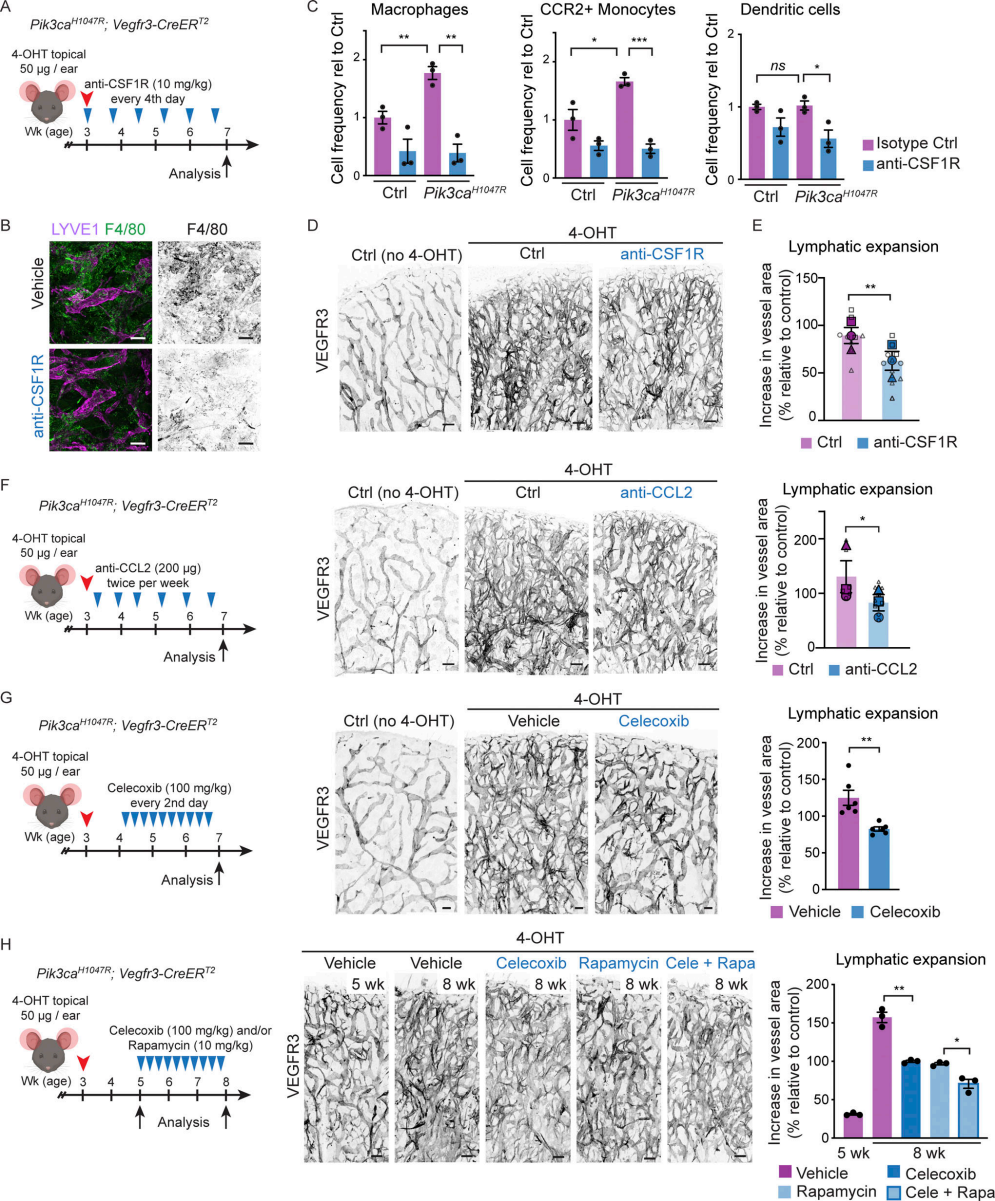
**Anti-inflammatory treatment limits *Pik3ca***^***H1047R***^**-driven lymphangiogenesis. (A)** Experimental scheme for depletion of monocyte/macrophage populations using anti-CSF1R antibody in the mouse model of *Pik3ca*^*H1047R*^-driven LM. **(B)** Whole-mount immunofluorescence of ears from a control (Ctrl) mouse, and 7-wk-old 4-OHT–treated *Pik3ca*^*H1047R*^;*Vegfr3-CreER*^*T2*^ mice following a 4-wk treatment with anti-CSF1R or vehicle, stained for myeloid marker F4/80. **(C)** Flow cytometry analysis of different myeloid cell populations in the ear skin of *Pik3ca*^*H1047R*^ mutants and littermate controls. Data represent cell frequency (of live cells) relative to isotype IgG-treated controls (*n* = 3 mice) ± SEM. **(D)** Whole-mount immunofluorescence of ears from A and B. **(E)** Quantification of lymphatic vessel area, shown as % increase in vessel area in comparison with untreated (no 4-OHT) control. Data from *n* = 8 (Ctrl) or 12 (anti-CSF1R) mice in three independent experiments (indicated by symbols) are represented in SuperPlot (mean ± SEM). **(F and G)** Left: Experimental scheme for anti-CCL2 (F) or celecoxib (G) treatment of *Pik3ca*^*H1047R*^ -driven LM. Middle: Whole-mount immunofluorescence of ears from a control (Ctrl) mouse, and 7-wk-old 4-OHT–treated *Pik3ca*^*H1047R*^;*Vegfr3-CreER*^*T2*^ mice following a 4-wk (F) or 2-wk (G) treatment period. Right: Quantification of lymphatic vessel area, shown as % increase in vessel area in comparison with control. Data in F from *n* = 8 (Ctrl) or 9 (anti-CCL2) mice in three independent experiments (indicated by symbols) are represented in SuperPlot (mean ± SEM). Data in G represent mean (*n* = 6 mice) ± SEM. **(H)** Left: Experimental scheme for extended celecoxib treatment of established *Pik3ca*^*H1047R*^-driven LM. Middle: Whole-mount immunofluorescence of ears from 4-OHT–treated *Pik3ca*^*H1047R*^;*Vegfr3-CreER*^*T2*^ mice at the start of the treatment period (5 wk), and following a 3-wk treatment with celecoxib and/or rapamycin, or vehicle (8 wk). Right: Quantification of lymphatic vessel area, shown as % increase in vessel area in comparison with the control. Data represent mean (*n* = 3 mice) ± SEM. P value in C and E–H: two-tailed unpaired Student’s *t* test; ***, P < 0.001; **, P < 0.01; *, P < 0.05; ns, P > 0.05. Scale bars: 100 μm (B, D, and F–H).

**Figure S5.**
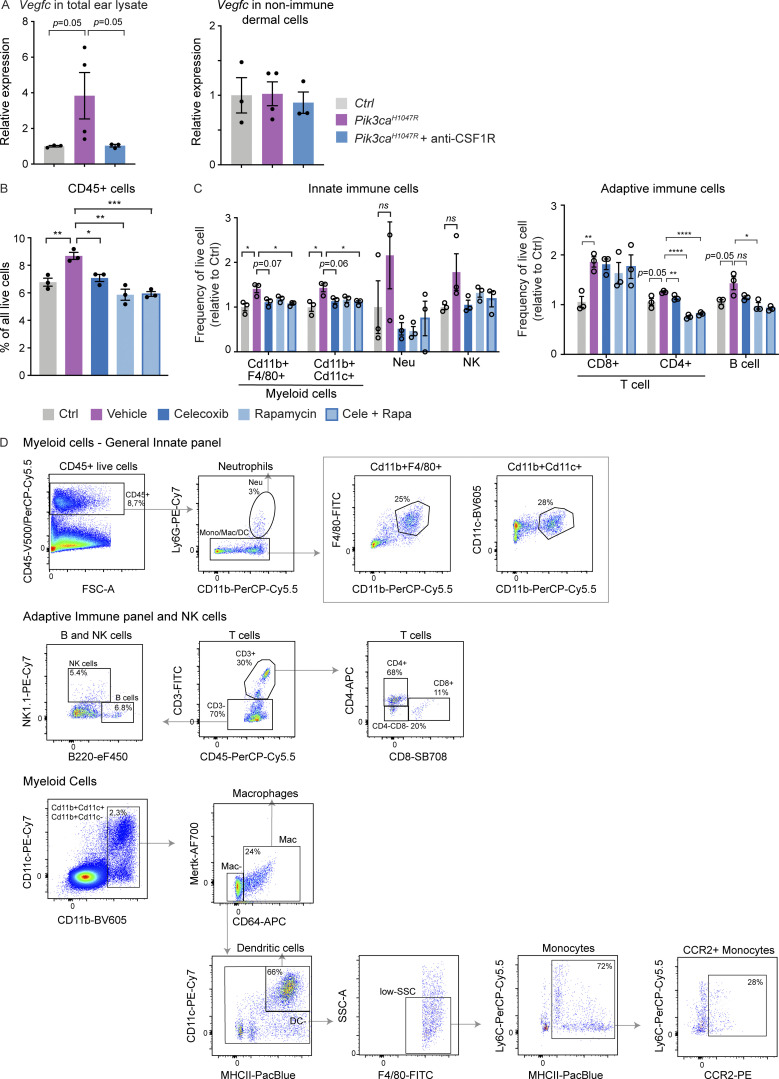
**Anti-inflammatory treatment in *Pik3ca***^***H1047R***^**-driven LM. (A)** qRT-PCR analysis of *Vegfc* in total ear skin lysate (on the left) and live non-immune dermal cell population (on the right; gated out all myeloid cells, neutrophils, NK cells, B cells, and T cells using panels shown in Fig. S5 D) from *Pik3ca*^*H1047R*^*; Vegfr3-CreER*^*T2*^ mice following treatment with anti-CSF1R or isotype control as in Fig. 9 A. Data represent mean relative expression (normalized to *Hprt*; *n* = 3–4 mice) ± SEM, presented relative to control mice. **(B and C)** Flow cytometry analysis of CD45^+^ cells (B), and subpopulations of innate and adaptive immune cells (C) in the ear skin of 4-OHT–treated 8-wk-old *Pik3ca*^*H1047R*^*; Vegfr3-CreER*^*T2*^ mice and littermate controls following treatment as in Fig. 9 H. Neu, neutrophil. Data represent relative cell frequency (of live cells) relative to the control (*n* = 3 mice) ± SEM. P value obtained with Mann–Whitney U test (A) or two-tailed unpaired Student’s *t* test (B and C). ****, P < 0.0001; ***, P < 0.001; **, P < 0.01; *, P < 0.05; ns, P > 0.05. Value P = 0.05 indicated on the graph. **(D)** Gating scheme for flow cytometry analysis of dermal immune cells. Percentage of gated cells in the gating scheme of General Innate panel: 8.7% of CD45^+^ live cells, of which 3% are neutrophils. The rest of the immune population (97%) is gated as 25% of Cd11b^+^F4/80^+^ and 28% of Cd11b^+^Cd11c^+^ myeloid cells. Percentage of gated cells in Adaptive Immune panel and NK cells: after gating the CD45^+^ live cells, similar to the General Innate panel, 30% of the cells are gated as CD3^+^ T cells and 70% as CD3^−^. Of the latter, 5.4% are gated as NK cells and 6.8% as B cells. The CD3^+^ T cells are additionally gated as 68% CD4^+^, 11% CD8^+^ and ∼20% double negative cells. Percentage of gated cells in myeloid cells panel: 2.3% CD11b^+^CD11c^−^ and CD11b^+^CD11c^+^ cells, of which 24% are gated as macrophages (Mac). 66% of non-Mac cells are dendritic cells (DC^+^). Non-DC low-SSC F4/80^+^ cells were further gated as 72% monocytes, of which 28% are CCR2^+^. The percentages in the shown example scheme vary depending on the age of the mouse and treatment.

Based on the increased lymphatic endothelial expression of immune-related molecules and pro-lymphangiogenic myeloid cell infiltrate, we hypothesized that paracrine LEC-myeloid cell crosstalk may critically contribute to promoting pathological vessel growth in the *Pik3ca*^*H1047R*^ mice. To inhibit the expansion and differentiation of macrophages (MacDonald et al., 2010), we administered a blocking antibody against the macrophage colony-stimulating factor 1 receptor (CSF1R) from the time of induction of *Pik3ca*^*H1047R*^ expression (Fig. 9 A). A 4-wk treatment period with anti-CSF1R antibody partially depleted F4/80^+^ myeloid cells (Fig. 9 B). Specifically, the frequency of macrophages and monocytes expressing the CCL2 receptor CCR2 were strongly reduced, while dendritic cells showed only a modest decrease (Fig. 9 C). Macrophage depletion inhibited the increase in the levels of *Vegfc* in the total lysate, but not in sorted non-immune dermal cells, from mutant in comparison with control ears (Fig. S5 A). This was associated with decrease in *Pik3ca*-driven lymphatic vessel growth (Fig. 9, D and E). To specifically inhibit CCL2-mediated recruitment and differentiation of CCR2^+^ monocytes, we treated mice with an antibody against CCL2 (Fig. 9 F). A comparable decrease in *Pik3ca*-driven lymphatic vessel expansion compared to the effect of CSF1R inhibition was observed (Fig. 9 F). Together, these results support that monocyte/macrophages account for the increased production of *Vegfc* in *Pik3ca* mutant ears and indicate their critical requirement for LM growth.

Monocyte-derived macrophages are associated with the formation of lymphoid aggregates and tertiary lymphoid organs (TLOs) that develop in response and contribute to non-resolving chronic inflammation (Luo et al., 2019). Interestingly, TLOs are frequently found in patients with LMs (Kirsh et al., 2011), suggesting that efficient treatment of advanced lesions requires targeting of a more complex inflammatory environment. Inhibition of cyclooxygenase-mediated production of prostaglandins, and in particular cyclooxygenase 2 (COX-2) using celecoxib, has a potent effect on both macrophage recruitment and cytokine release (Hosono et al., 2016; Majumder et al., 2014), but also an immunosuppressive effect on T cells (Iñiguez et al., 1999) that we found to be increased in advanced LM lesions in mice. To test the therapeutic effect of COX-2 selective inhibition on LM progression, we administered celecoxib to *Pik3ca*^*H1047R*^;*Vegfr3-CreER*^*T2*^ mice 10 d after induction of lymphatic vessel overgrowth (Fig. 9 G). Systemic COX-2 inhibition significantly reduced vessel growth after a 2-wk treatment period (Fig. 9 G). Notably, the 34% reduction in *Pik3ca*-driven vascular expansion upon celecoxib treatment is comparable to the effect of the mTOR inhibitor rapamycin reported after a 1.5-wk treatment period in this model (Martinez-Corral et al., 2020).

To assess the effects of celecoxib and rapamycin on more advanced lesions and their immune cell infiltration, we administered them alone or in combination 2 wk after induction of lymphatic vessel overgrowth and analyzed the mice after a 3-wk treatment period (Fig. 9 H). Individually administered inhibitors showed a similar 60% reduction in vascular expansion, and an additive effect with 87% reduction when used in combination (Fig. 9 H). FACS analysis of the ear skin of celecoxib-treated *Pik3ca* mutant mice showed a decrease in the frequency of CD45^+^ cells (Fig. S5 B). Notably, a similar effect was observed after treatment with rapamycin (Fig. S5 B). Increase in CD45^+^ cells, and specifically myeloid cells, was prominently inhibited by a combination treatment (Fig. S5, B and C), while celecoxib and rapamycin did not affect CD3^+^CD8^+^ T cell numbers (Fig. S5 C). Other lymphocyte populations including CD3^+^CD4^+^ T cells and B cells were only modestly increased in the mutant skin at this stage, and more prominently reduced by rapamycin compared to celecoxib (Fig. S5 C).

Collectively, our data show that inhibition of CSF1R, CCL2, or COX-2 limit *Pik3ca*-driven lymphangiogenesis, suggesting that anti-inflammatory therapy provides a potential therapeutic approach for the treatment of LM. Our results also suggest that in addition to inhibiting the downstream PI3K target mTOR in LECs, the immune suppressive effect of rapamycin affecting both myeloid and lymphoid cells may contribute to its therapeutic benefit in the treatment of LM.

## Discussion

Using a mouse model of oncogenic PI3K-driven VMs and LMs, we characterized vessel type–specific pathogenic responses to the common causative *Pik3ca*^*H1047R*^ mutation. We uncover a new immune-interacting subtype of dermal lymphatic capillary ECs, iLECs, that reside at capillary terminals in normal vasculature and selectively increase in number in the *Pik3ca*^*H1047R*^-driven LM. Increased expression of pro-inflammatory factors by iLECs and associated recruitment of VEGF-C–producing macrophages in turn support pathological lymphangiogenesis that is inhibited by targeting of the associated immune response.

*PIK3CA* mutations specifically cause vascular malformations in veins and lymphatic vessels. The basis of such vessel type–restricted disease manifestation is unknown. We found that the activation of oncogenic p110α-PI3K signaling in the embryonic or postnatal vasculature triggered distinct cellular responses in different vessel types, characterized by vessel sprouting (lymphatic vessels), localized dilation (blood capillaries and veins), or no apparent effect (arteries). Why the same oncogenic stimulus induces different responses in these cells remains unclear, but likely both EC-autonomous and non-autonomous mechanisms play a role.

Modeling of *Pik3ca*-driven VMs in the mouse retina recently uncovered that active angiogenesis is required for vascular overgrowth (Kobialka et al., 2021), similar to what has been reported in other types of vascular malformations (Ola et al., 2016; Park et al., 2009; Boulday et al., 2011; Bravi et al., 2015). Here, we observed a similar vascular response to *Pik3ca*^*H1047R*^ expression in growing embryonic as well as quiescent postnatal dermal blood and lymphatic vessels. Tissue-specific differences in the availability of growth factors that can synergize with the oncogenic p110α-PI3K to enhance downstream signaling (Martinez-Corral et al., 2020) may provide a potential explanation for the apparently different vascular responses in the postnatal retina and skin.

In agreement with previous observations (Castillo et al., 2016; Martinez-Corral et al., 2020; Kobialka et al., 2021), proliferation was an early response of both venous ECs and LECs to oncogenic *Pik3ca*. However, proliferation was sustained selectively in LECs of advanced lesions. The first weeks of lymphatic, but not blood vessel, overgrowth was associated with a selective increase in the abundance of macrophages and CCR2^+^ monocytes. The latter is particularly noteworthy, given the low abundance of monocytes in healthy tissues and their recruitment from the blood to the site of inflammation, where they differentiate into proinflammatory macrophages (Gordon and Taylor, 2005; Italiani and Boraschi, 2014). Chemokines and cytokines associated with the recruitment of monocytes (CCL2/MCP1; Italiani and Boraschi, 2014) and their proinflammatory signaling (IL1β, IL6, and TNFα; Viola et al., 2019) were also increased. Importantly, we found that macrophages from the mutant mice showed increased expression of the pro-lymphangiogenic growth factor VEGF-C, which we previously found to be required for *Pik3ca*-driven LM growth (Martinez-Corral et al., 2020). In further support of a role of monocytes/macrophages in promoting LM progression, we found that the blockade of their recruitment or differentiation using anti-CSF1R or anti-CCL2/MCP1 antibodies inhibited *Pik3ca*-driven lymphatic overgrowth. Although our study did not assess a causal link between macrophage recruitment, VEGF-C production, and lymphangiogenesis in a wild-type setting, previous studies in different disease contexts have established such links (Schoppmann et al., 2002; Alishekevitz et al., 2016; Glinton et al., 2022; Kataru et al., 2009). Of note, the recruited macrophages may additionally produce factors that promote proteolytic processing of, or synergize with VEGF-C, to enhance its lymphangiogenic activity (Jha et al., 2017).

**Figure 10.**
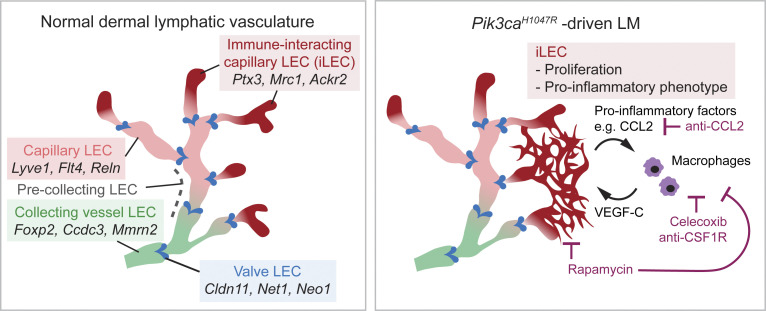
**Proposed model of paracrine LEC-immune cell interactions as a driver of LMs.** Schematic illustration showing distinct dermal LEC subtypes within lymphatic vessel hierarchy (on the left), and pathological changes driven by oncogenic *Pik3ca*^*H1047R*^ (on the right). *Pik3ca*^*H1047R*^ drives expansion and activation of a pro-inflammatory transcriptome in iLECs to induce recruitment and activation of myeloid cells that in turn promote pathological lymphangiogenesis by secretion of pro-lymphangiogenic factors. Celecoxib and anti-CSF1R or anti-CCL2 therapy inhibit lymphangiogenesis by reducing myeloid cell numbers.

LECs have been shown to express certain chemokines and cytokines, but their role in direct paracrine activation of myeloid cells has not been explored. Using single-cell transcriptomics, we identified a dermal LEC hierarchy that recapitulates the lymphatic vascular architecture of collecting vessels and capillaries in vivo (Fig. 10). This analysis additionally identified a previously unknown and molecularly distinct *Ptx3*^*high*^ population within dermal lymphatic capillary terminals as an immune-interacting LEC subtype, termed iLEC. The iLEC population shared features of a distinct *Ptx3*^*+*^ lymph node LEC subset (Xiang et al., 2020) and was characterized by high expression of transcripts encoding key regulators of innate and adaptive immune responses (e.g., *Ptx3*, *Mrc1*, *Ackr2*). We observed selective expansion and proliferation of the *Ptx3*^*high*^ iLECs in the *Pik3ca*^*H1047R*^ mice, and high lymphatic endothelial expression of PTX3 in *PIK3CA*^*H1047R*^-driven LM in humans. Additional upregulation of pro-inflammatory genes, including *Ccl2*, in iLECs suggested that their direct role in paracrine myeloid cell recruitment and activation, as well as the pathological cell population in LM. Interestingly, lymph node LECs can present antigens to regulate T cell fate and function (reviewed in Santambrogio et al., 2019; Lucas and Tamburini, 2019), and similar immunoregulatory functions of LECs were recently reported outside of the LN in the context of neuroinflammation and cancer (Hsu et al., 2022; Gkountidi et al., 2021; Lane et al., 2018). Our finding of the dermal iLEC subtype of peripheral lymphatic vessels raises a possibility that LECs have wider roles in the regulation of the immune response, even under homeostasis.

In addition to producing lymphangiogenic growth factors, monocyte-derived macrophages are associated with the formation of lymphoid aggregates and TLOs in chronic inflammation (Luo et al., 2019), that are frequently found in patients with LMs (Kirsh et al., 2011). Targeting of a broader immune response, e.g., by inhibiting the production of prostaglandin, may thus be required for efficient treatment of advanced lesions. We found that systemic COX-2 inhibition using celecoxib limited *Pik3ca*^*H1047R*^-driven lymphangiogenesis in mice. Inhibition of macrophage recruitment (our study and Hosono et al., 2016; Majumder et al., 2014) or VEGF-C/D production (Majumder et al., 2014; Lyons et al., 2014; Kashiwagi et al., 2011) by celecoxib may account for its anti-lymphangiogenic effects. However, other mechanisms including its direct effects on the immune functions of LECs themselves (Christiansen et al., 2016) or T cells (Iñiguez et al., 1999) cannot be excluded. Notably, one patient with intractable progressively growing cervical LM was successfully treated with celecoxib (Imamura et al., 2019). Although not curative, celecoxib or other anti-inflammatory treatments could thus provide a clinical benefit in patients with progressive LM.

The mTOR inhibitor rapamycin has provided beneficial effects in clinical trials for VM and LM treatment (Queisser et al., 2021; Mäkinen et al., 2021). Because rapamycin induces cell-cycle arrest, sustained proliferation of LECs in advanced LM lesions may make them exquisitely sensitive to rapamycin. Rapamycin can exert additional inhibitory effects on LM growth by reducing VEGFR3 levels and thereby upstream lymphangiogenic growth factor signaling in LECs (Luo et al., 2012; Baluk et al., 2017). VEGFR3 downregulation is also observed in mice lacking *Pik3ca* in LECs, and upon treatment with other PI3K pathway inhibitors (Korhonen et al., 2022). Our results further indicate that rapamycin affects the recruitment of both myeloid and lymphoid cells, which may additionally contribute to its therapeutic benefit in the treatment of LM. Given the multiple effects of PI3K inhibition on LM pathology, as data from clinical trials become available, it will be of interest to investigate whether rapamycin or other PI3K pathway inhibitors provide a better therapeutic outcome in the treatment of *PIK3CA*-driven LMs compared to VMs.

Taken together, our study reveals a new immune-interacting subtype of dermal lymphatic capillary ECs, iLECs, as a pathological cell population in LM. These iLECs produce factors for the recruitment of VEGF-C–expressing macrophages that in turn support pathological lymphangiogenesis. Apart from identifying the immune response as a therapeutic target for the treatment of LM, our findings have implications for understanding the role of lymphatic vessels as upstream orchestrators of the immune response in other inflammatory conditions.

## Materials and methods

### Mouse lines and treatments

*R26-LSL-Pik3ca*^*H1047R*^ (Eser et al., 2013), *R26-mTmG* (Muzumdar et al., 2007), and *tdTom* (Madisen et al., 2010) mice were crossed with the *Cdh5-CreER*^*T2*^ (Wang et al., 2010), *Vegfr3-CreER*^*T2*^ (Martinez-Corral et al., 2016), or *Vegfr1-CreER*^*T2*^ (generated in this study; Fig. S1 A). All mice were analyzed on a C57BL/6J Jax background (backcrossed minimum four times). Both female and male mice were used for analyses and no differences in the phenotype between the genders were observed. The morning of vaginal plug detection was considered as E0. Cre-mediated recombination was induced in embryos by intraperitoneal injection of 0.5 mg (*Cdh5-CreER*^*T2*^) or 2 mg (*Vegfr1-CreER*^*T2*^, *Vegfr3-CreER*^*T2*^) of 4-OHT (H7904; Sigma-Aldrich), dissolved in peanut oil (10 mg/ml), to pregnant females at E10 (*Vegfr1-CreER*^*T2*^), E11 (*Cdh5-CreER*^*T2*^, *Vegfr3-CreER*^*T2*^), or at E14 (*Cdh5-CreER*^*T2*^). For postnatal induction, 25 μg (*Cdh5-CreER*^*T2*^) or 50 μg (*Vegfr3-CreER*^*T2*^ and *Vegfr1-CreER*^*T2*^) of 4-OHT dissolved in acetone (10 mg/ml) was administered topically to the dorsal side of each ear of 3-wk-old mice. Littermate controls were included in each experiment, which were either Cre^−^ mice treated with 4-OHT (*Cdh5-CreER*^*T2*^, *Vegfr1-CreER*^*T2*^), or Cre^+^ mice treated with the vehicle (*Vegfr3-CreER*^*T2*^). 4-OHT–treated mice carrying the different Cre alleles in combination with the *R26-mTmG* reporter showed normal vasculature, excluding Cre-induced toxicity (Brash et al., 2020) as the cause of the phenotypes in the *Pik3ca*-expressing mice. Celecoxib (C-1502; LC Laboratories) was dissolved first in DMSO (400 mg/ml) followed by mixing 1:20 with oil (final concentration of 20 mg/ml) and administered by oral gavage every second day at the dose of 100 mg/kg. InVivoMAb anti-mouse CSF1R antibody (clone AFS98, BE0213; BioXCell) was diluted in PBS (experiment 1) or in InVivoPure pH 7.0 Dilution Buffer (IP0070; BioXCell; experiment 2 and 3) and administered by intraperitoneal injection (10 mg/kg) twice per week. Control mice were administered with the vehicle (experiment 1) or isotype control (InVivoMAb rat IgG2a isotype control, anti-trinitrophenol, clone 2A3, BE0089; BioXCell; experiment 2 and 3). InVivoMab anti-human/mouse CCL2 (MCP-1) antibody (clone 2H5, BE0185; BioXCell) was diluted in InVivoPure pH 7.0 Dilution Buffer (IP0070; BioXCell) and administered by intraperitoneal injection (200 µg) twice per week. Control mice were administered with Isotype Control (InVivoMAb polyclonal Armenian hamster IgG, BE0091; BioXCell).

### Generation of *Vegfr1-CreER*^*T2*^ mice

To generate transgenic mice expressing the tamoxifen inducible *CreER*^*T2*^ under the control of the *Flt1* (*Vegfr1*) promoter, a BAC (B6Ng01-247E8) carrying the mouse *Vegfr1* gene (RefSeq NM_010228.3) was obtained from the mouse B6Ng01 library. The BAC vector was engineered to replace the coding sequence in exon 1 as well as the splice donor site at the junction between exon 1 and intron 1 (50 bp) with a cassette containing the open reading frame for *CreER*^*T2*^ and the *Vegfr1* 3′ untranslated region (UTR), such that the endogenous translation initiation codon from the *Vegfr1* gene is used for the expression of the CreER^T2^ protein. A polyadenylation signal (human growth hormone polyadenylation signal) was inserted 3′ of the *Vegfr1* 3′UTR sequence (Fig. S1 A). The engineered BAC vector was used for pronuclear injection into fertilized C57BL/6NTac oocytes. Two independent founder lines were generated, of which one showed efficient Cre-mediated recombination in the *Vegfr1*-expressing cells and was used for further analyses. Mice were generated by Taconic Biosciences.

Transgenic mice were detected by PCR with primers designed to amplify a 494 base pair region at the junction of 5′ mouse genomic region and the *CreER*^*T2*^ open reading frame in the transgene (forward 19966_7: 5′-CAC​TTC​AGC​GAG​GTC​CTT​GAG-3′ 5 and reverse 19966_112: 5′-CAT​CTT​CAG​GTT​CTG​CGG​G-3′). Additional control primers (forward 11767_3: 5′-GGG​GCA​ATC​AAT​TGA​GGG-3′ and reverse 11767_4: 5′-CAA​CCT​CTG​CTT​GGT​TCT​GG-3′) were included in the reaction to amplify a 333 bp fragment. Standard amplification reactions (25 μl) were prepared using 0.4 μM of each primer and 0.2 mM deoxynucleotide triphosphates. After initial denaturation at 95°C for 5 min, reactions were subjected to 35 cycles of 95°C (30 s), 60°C (30 s), and 72°C (60 s). Reactions were incubated for a final elongation at 72°C for 10 min. PCR products were separated on a 2% agarose gel containing Sybr Safe.

### Antibodies

The details of primary antibodies used for immunofluorescence of whole mount tissues and flow cytometry are provided in Table S2. Secondary antibodies conjugated to AF488, AF594, AF647 or Cy3 were obtained from Jackson ImmunoResearch and used in 1:300 dilution (Table S2).

### Whole-mount immunofluorescence

Tissues (skin, diaphragm, or intestine wall) were fixed in 4% paraformaldehyde for 2 h at room temperature and permeabilized in 0.3% Triton X-100 in PBS (PBST) for 10 min. After blocking in PBST with 3% milk for at least 1.5 h, the tissues were incubated with primary antibodies at 4°C overnight in blocking buffer, followed by PBST washing and incubation with fluorescence-conjugated secondary antibodies for 2 h at room temperature. After washes in PBST, the samples were mounted in Mowiol. PTX3 and CCL2 staining was amplified by Tyramide Signal Amplification kit (TSA, NEN Life Science Products). Tissue was first blocked with TNB (Tris-NaCl–blocking buffer), prepared according to the TSA kit instructions. For CCL2 staining the rest of the protocol was done according to TSA kit instructions with PBST used as a permeabilization reagent. PTX3 staining and washing of the tissue was done in PBS in the absence of permeabilization agent to allow visualization of extracellular proteins.

### EdU Click-iT Kit assay

DNA synthesis in proliferating cells was detected using the Click-iT EdU Cell Proliferation Kit for Imaging (Thermo Fisher Scientific). Mice received an intraperitoneal injection with 25 mg/kg of EdU 16 h prior to harvesting the ears for analysis. After whole-mount immunofluorescence staining (specified above, except for PROX1 staining that was performed after the Click-iT assay), the tissues were washed extensively with PBST and stained for EdU according to the manufacturer’s instructions. Briefly, tissues were incubated in the Click-iT Reaction cocktail for 40 min at room temperature followed by washing in PBST.

### Confocal microscopy and image processing

All confocal images were acquired using a Leica SP8 or a Leica STELLARIS 5 confocal microscope equipped with a white light laser and 10×/0.45 C-Apochromat (HC PL APO CS2), 20×/0.75 (HC PL APO CS2), 25×/0.95 (HC FLUOTAR L VISIR), or 63×/1.20 (HC PL APO) objective. The images were obtained at room temperature using Leica LAS X software. All images were processed using Fiji ImageJ (National Institutes of Health) software. Each image represents maximum intensity projection of a Z-stack (capturing the entire lymphatic vascular layer, or the whole tissue Z-stack when imaging immune populations) of single tiles or multiple tile scan images. The ear tile scans in Fig. S1 were obtained using DMI8 Leica fluorescence microscope (Fig. S1 G) or Leica Thunder Imaging System (Fig. S1 H). The close-up images in Figs. 2, D and E; 3, A and C; 5 E; 6 H; 8 D; S2 F; and S4, E and F were additionally deconvolved using Huygens Essential (version 19.04; Scientific Volume Imaging) with theoretical point spread function and automatic background estimation. Details of image processing and quantification are provided in the supplemental material.

### FACS analysis

FACS analysis of Ki67^+^ ECs and CD45^+^CD11b^+^F4/80^+^ cells was done as previously described (Martinez-Corral et al., 2020). For FACS analysis of innate and adaptive immune cells, the ear skin was dissected, cut into pieces, and digested in Liberase TL (100 µg/ml; Roche; for innate immune cells) or Collagen IV (2 mg/ml; Life Technologies; for adaptive and NK cells) plus DNase I (0.5 mg/ml; Roche) in PBS with 0.2% FBS at 37°C for 2 h at 700 rpm. The cell lysate was filtered through 50 µm filters (CellTrics, Sysmex) and washed with FACS buffer. The cells were first incubated with rat anti-mouse CD16/32 antibody (eBioscience) to block Fc receptor binding. Cell suspensions were stained for antibodies targeting different immune populations (Table S2). Dead cells were labeled using LIVE/DEAD Fixable Near-IR Dead Cell Stain Kit (Life Technology). The analysis was performed on BD LSRFortessa Cell Analyser (BD Biosciences). All data were processed using FlowJo software version 10.5.0 (TreeStar). Gating of Ki67^+^ ECs and CD45^+^CD11b^+^F4/80^+^ was done as previously described (Martinez-Corral et al., 2020). Gating schemes for general innate and adaptive immune panels, as well as distinct myeloid populations (macrophages, dendritic cells and monocytes) are shown in Fig. S5 D. Relative cell frequency of the subtypes of immune populations is presented as fold change (in % of all live cells) relative to the average of the control in each experiment. The absolute cell numbers and cell frequencies presented as % of all live cells are provided in Data S1.

### Single-cell transcriptomics

Dermal LECs and BECs were FACS-sorted from the ear skin of 4-OHT–treated 5-wk-old *Pik3ca*^*H1047R*^;*Cdh5-CreER*^*T2*^ (*n* = 5) and Cre^−^ littermate control mice (*n* = 2) of mixed genders. This stage was chosen as the earliest timepoint showing robust vascular overgrowth, high LEC proliferation and immune cell infiltration in the mutants. One wild-type C57BL/6J mouse, not treated with 4-OHT, was also included, to exclude possible effect of the solvent and 4-OHT on EC transcriptome. Ear skin was first digested in 5 mg/ml Collagenase II in PBS supplemented with 0.2 mg/ml DNaseI and 0.2% FBS, followed by filtering through 50 µm filters (CellTrics, Sysmex). Fc-receptors were blocked with rat anti-mouse CD16/32 antibody (eBioscience), followed by staining using Podoplanin-APC and CD31-Pe-Cy7 antibodies. Dump channel included erythrocytes and immune cells (labeled using CD45-eF450, CD11b-eF450 and Ter119-eF450) and dead cells (SYTOX Blue dead stain; Life Technology). The sorting into 384-well plates was performed with a 100 µm nozzle on BD FACS AriaIII CellSorter (BD BioScience Flow Cytometry System equipped with four lasers: 405, 488, 561, 633 nm). Smart-Seq2 library preparation and sequencing were performed as described previously (Picelli et al., 2014). Key quality metrics are listed in Table S3.

### scRNA-seq data processing

The single-cell sequence data were aligned to the mouse reference genome GRCm38 with tophat (version 2.1.1; Kim et al., 2013) and *Gallus gallus PIK3CA* sequence (National Center for Biotechnology Information [NCBI] nucleotide sequence ID: NM_001004410). The latter was used to identify the *Pik3ca*^*H1047R*^ transcript in the transgenic mice, encoded by *G. gallus Pik3ca*, which is highly homologous (83%) to the mouse *Pik3ca* (NM_008839.3). The corresponding protein sequence of *G. gallus* (NP_001004410) is 96% identical to mouse (NP_032865.2), and oncogenic in mammalian cells (Eser et al., 2013; Bader et al., 2006). Duplicated reads were filtered out using samtools software (version 0.1.18). The gene counts were summarized using featureCounts function from the Subread package (version 1.4.6-p5; Liao et al., 2014). Further downstream analysis of raw expression data was performed in RStudio (desktop version 2021.09.2 + 382) using R versions 3.5.1 and 4.0.3 (Satija et al., 2015; Butler et al., 2018). The following quality control steps were performed: (1) genes expressed by fewer than three cells were removed; (2) cells with fewer than 200 genes or 50,000 reads counts were not further considered; (3) cells in which over 10% reads derived from the mitochondrial genome were removed. After removing cells with poor transcriptome quality, the data were processed in Seurat package (version 3.1.1 and 4.0.1; Hao et al., 2021; Stuart et al., 2019) for normalization using *LogNormalize* function, graph-based clustering analysis (Louvain method), non-linear dimensional reduction (UMAP) and DEG detection for identification of cluster markers (Wilcoxon rank sum test, marker genes selected by P value with Bonferroni correction <0.05 and logarithmic fold change >1). The data were further processed in two steps in which an LEC population was extracted from the control (including Cre^−^ littermates and wild-type C57BL/6J) and mutant datasets individually after removal of contaminating cells identified as epithelial cells/keratinocytes, fibroblasts, mural cells and immune cells based on DEG analysis (Data S9). In the second step, LECs from the control and mutant mice were integrated, and additional small clusters with neuronal, immune cell, and fibroblast identities were removed (Data S9). Batch correction and data integration was performed using Harmony package (version 1.0; Korsunsky et al., 2019) for mutant and integrated control/mutant data. Finally, additional dermal LECs from two 4-wk-old Cre^−^ mice, published previously under Gene Expression Omnibus accession no. GSE202989 (Ctrl_1 and Ctrl_4; Korhonen et al., 2022), were integrated with control LECs using the canonical correlation analysis method, and assessed for contaminants as described above.

#### Trajectory inference analysis

Trajectory inference analysis was performed using *SCORPIUS* (version 1.0.5; Cannoodt et al., 2016 *Preprint*) for the control dataset (Fig. 5 B) and *SLINGSHOT* (version 2.2.0; Street et al., 2018) for the combined dataset (Fig. 6 D) in which the respective algorithm constructed the topology of dynamic process as a linear trajectory and mapped the cells along this trajectory curve. In the process of constructing the trajectory using *SCORPIUS*, we used *k* = 5 and other parameters as default. Default parameters of *SCORPIUS* (but number of threads = 10) were applied to calculate feature importance to predict ordering of genes along the trajectory. Resulting genes of importance were visualized in a heatmap ranked by their position in the along the trajectory. Using *SLINGSHOT*, two lineages/branches were obtained by constructing minimum spanning trees on clusters in an unsupervised manner using default parameters without forcing a cluster of origin or leaf node.

#### GO analysis

For GO enrichment analysis on the biological processes domain, the hypergeometric test in the *GOstats* package (version 2.56.0) was applied. The analysis was restricted to gene sets containing 5–1,000 genes. Significant pathways were filtered by applying P value <0.05 and gene count/term >10. Further selection of relevant GO terms was based on sorting terms on their OddsRatios (the ratio of a GO term in the differently expressed genes list to the occurrence of this GO term in a universal gene list, obtained from org.Mm.eg.db [version 3.13.0]). In addition, GO term results were screened for enrichment of terms related to immune regulation. Selected top pathways were visualized using ggplot2 (version 3.3.5).

A web application for data searching and visualization was generated using the shiny package of Rstudio (https://shiny.rstudio.com), and the package ShinyCell for database creation (Ouyang et al., 2021).

### RNA extraction and real-time qPCR

Total RNA was extracted from FACS-sorted BECs and LECs, or CD45^+^CD11b^+^Ly6G^−^F4/80^+^ myeloid cells, macrophages (Cd11b^+^MerTK^+^CD64^+^) and dendritic cells (MerTK^−^CD64^−^CD11c^+^MHCII^+^), using RNeasy Micro Kit (QIAGEN), according to the manufacturer’s instructions with additional DNaseI treatment (RNAse-Free DNase Set, QIAGEN). The cDNA was synthesized using Superscript VILO Master Mix (Invitrogen) and TaqMan PreAmp Master Mix was used for preamplification with subsequent real-time qPCR on StepOne Plus Real-Time PCR system (Applied Biosystems) using TaqMan gene expression Master Mix (Applied Biosystems). Total RNA from primary dermal LECs, isolated from the *Pik3ca*^*H1047R*^;*Vegfr3-CreER*^*T2*^ mice and treated with 4-OHT as previously described (Martinez-Corral et al., 2020), was extracted and processed the same way. The following TaqMan Assays were used: *Hprt* (Mm01545399_m1), *Mki67* (Mm01278617_m1), *Vegfc* (Mm00437310_m1), *Prox1* (Mm00435969_m1), and *Ccl2* (Mm00441242_m1). Relative gene expression levels were calculated using the comparative CT method with *Hprt* as reference gene.

### Multiplex ELISA

Meso Scale Diagnostics (MSD) Multiplex ELISA using MSD Multi-Spot Assay System was used with two different V-PLEX MSD cytokine assays for detection of total 19 proteins, divided into two panels—Proinflammatory Panel 1 (including IFN-γ, IL-1β, IL-2, IL-4, IL-5, IL-6, CXCL1 [KC/GRO], IL-10, IL-12p70, TNF-⍺) and Cytokine Panel 1 (IL-9, MCP-1, IL-33, IL-27p28/IL-30, IL-15, IL-17A, MIP-1a, IP-10, MIP-2). Ear skin lysates were prepared by homogenizing the tissue with Homogenizer TissueRuptor II (QIAGEN) in TBS + 1% Triton X-100 + Protease and Phosphatase Inhibitor Cocktail (Sigma-Aldrich). Protein concentration was measured and adjusted to 100 mg/ml before adding the samples to 96-well plate-based multiplex assay plate (provided by Meso Scale Discovery Kit) containing detection antibodies, conjugated with electrochemiluminescent labels (MSD SULFO-TAG). Subsequent steps were performed according to the manufacturer’s instructions. Samples which gave a readout below the manufacturer’s control threshold were excluded.

### Analysis of human tissue

Human biopsy material was obtained from Xarxa de Bancs de Tumors de Catalunya biobank, approved by PIC-96-16. Histology was examined by a pathologist expert in vascular anomalies and summarized in Table S1. PTX3 staining of paraffin sections was amplified by a TSA kit (NEN Life Science Products). Tissue was blocked with TNB (Tris-NaCl–blocking buffer) and prepared according to the TSA kit instructions. Samples were mounted using VECTASHIELD HardSet Antifade Mounting Media (Vector Laboratories).

### Statistics

Graphpad Prism 7–9 was used for graphic representation and statistical analysis of the data. A Shapiro–Wilk normality test was used to assess whether or not the data between two groups is normally distributed. When the data were normally distributed, unpaired two-tailed Student’s *t* test was used (with or without additional Welch’s correction after performed F-test for equal variances). When the data were not normally distributed, Mann–Whitney U test was used instead. The exact statistical test used is added to the figure legend. Differences were considered statistically significant when P *<* 0.05 and indicated on the graphs with star symbols: ****, P *<* 0.0001; ***, P *<* 0.001; **, P *<* 0.01; *, P *<* 0.05; and ns, P *>* 0.05. Enrichment of GO terms between clusters was assessed using hypergeometric tests. GO term sizes were set to contain minimum 5 and maximum 1,000 members of the mouse genome. For identifying cluster markers from scRNA-seq data, Wilcoxon rank sum test was performed. Marker genes were selected by P value with Bonferroni correction <0.05 and logarithmic fold change >1.

### Study approval

Experimental procedures on mice were approved by the Uppsala Animal Experiment Ethics Board (permit numbers 130/15, 5.8.18-06383/2020) and performed in compliance with all relevant Swedish regulations. Human biopsy material was obtained from Xarxa de Bancs de Tumors de Catalunya biobank, approved by PIC-96-16. Analysis of human biopsy material was approved by Swedish Ethical Review Authority (Etikprövningsmyndigheten, permit number: Dnr 2020-00987).

### Online supplemental material

Fig. S1 shows generation of BAC transgenic *Vegfr1-CreER*^*T2*^ mice. Fig. S2 shows characterisation of a model of progressive vascular overgrowth in the *Pik3ca*^*H1047R*^*; Vegfr3-CreER*^*T2*^ and *Pik3ca*^*H1047R*^*; Vegfr1-CreER*^*T2*^ mice. Fig. S3 shows analysis of inflammatory cells and markers in *Pik3ca*-driven vascular lesions. Fig. S4 shows characterization of cell clusters in scRNA-seq datasets of *Pik3ca*^*H1047R*^ mutant and control LECs. Fig. S5 shows anti-inflammatory treatment in *Pik3ca*^*H1047R*^-driven LM. Table S1 shows clinical features of patients with LM driven by *PIK3CA*^*H1047R*^ mutation. Table S2 lists antibodies. Table S3 shows key quality metrics for dermal EC scRNA-seq data. Data S1 shows immune cell frequencies and cell counts for FACS data. Data S2 shows cluster gene markers for dermal LECs. Data S3 shows zonation markers for dermal LECs. Data S4 shows cluster gene markers for dermal LECs from the *Pik3ca*^*H1047R*^;*Cdh5-CreER*^*T2*^ mice. Data S5 shows cluster gene markers for dermal LECs from control and *Pik3ca*^*H1047R*^;*Cdh5-CreER*^*T2*^ mice. Data S6 shows DEGs between non-proliferative *Ptx3* capillary LECs from *Pik3ca*^*H1047R*^ mutant in comparison with *Ptx3* capillary LECs from control mice. Data S7 shows GO enrichment analysis of DEGs in non-proliferative *Ptx3* metabolic capillary LECs from *Pik3ca*^*H1047R*^ mutant in comparison with *Ptx3* capillary LECs from control mice. Data S8 shows GO enrichment analysis of DEGs in non-proliferative *Ptx3* LECs from *Pik3ca*^*H1047R*^ mutant and control mice. Data S9 shows marker genes of clusters removed during processing of LEC dataset from *Pik3ca*^*H1047R*^ mutant and control mice.

## Supplementary Material

Table S1 shows clinical features of patients with LM driven by *PIK3CA*^*H1047R*^ mutation.

Table S2 list antibodies.

Table S3 shows key quality metrics for dermal EC scRNA-seq data.

Data S1 shows immune cell frequencies and cell counts for FACS data. Immune cell frequencies presented as percentage of all live cells and cell counts of the immune subpopulations, as well as total cell count of live cells are included.

Data S2 shows cluster gene markers for dermal LECs defined by comparing cells in each cluster with all other cells in the other clusters. Wilcoxon rank sum test was used for identifying the gene markers (Bonferroni adjusted P value <0.05 and logarithmic fold change >1).

Data S3 shows zonation markers for dermal LECs. Zonation markers which contribute to predict LEC trajectory were identified by Random Forest regression model as described in the Materials and methods section.

Data S4 shows cluster gene markers for dermal LECs isolated from the *Pik3ca*^*H1047R*^;*Cdh5-CreER*^*T2*^ mice, defined by comparing cells in each cluster with all other cells in the other clusters. Wilcoxon rank sum test was used for identifying the gene markers (Bonferroni adjusted P value <0.05 and logarithmic fold change >1).

Data S5 shows cluster gene markers for dermal LECs from the control and *Pik3ca*^*H1047R*^;*Cdh5-CreER*^*T2*^ mice, defined by comparing cells in each cluster with all other cells in the other clusters. Wilcoxon rank sum test was used for identifying the gene markers (Bonferroni adjusted P value <0.05 and logarithmic fold change >1).

Data S6 shows DEGs between non-proliferative *Ptx3* capillary LECs from *Pik3ca*^*H1047R*^ mutant in comparison with *Ptx3* capillary LECs from control mice.DEGs were selected by P value with Bonferroni correction less than 0.05 and logarithmic fold change > 0.5 or < −0.5. Cells with *Ptx3* expression value greater than its third quantile were considered as *Ptx3*^+^ cells in control mice.

Data S7 shows GO enrichment analysis of DEGs in non-proliferative *Ptx3* metabolic capillary LECs from *Pik3ca*^*H1047R*^ mutant in comparison with *Ptx3* capillary LECs from control mice. GO enrichment analysis on the “biological processes” domain was performed using the hypergeometric test in the *GOstats* package (version: 2.56.0). The analysis was restricted to gene sets containing 5–1,000 genes. Significant pathways were selected with P value <0.05. Terms with >10 counts were included, and the table was sorted by OddsRatio and then by P value.

Data S8 shows GO enrichment analysis of DEGs in non-proliferative *Ptx3* LECs from *Pik3ca*^*H1047R*^ mutant and control mice. GO enrichment analysis on the “biological processes” domain was performed using the hypergeometric test in the *GOstats* package (version 2.56.0). The analysis was restricted to gene sets containing 5–1,000 genes. Significant pathways were selected with P value <0.05. Terms with >10 counts were included, and the table was sorted by OddsRatio and then by P value.

Data S9 shows marker genes of clusters removed during processing of LEC dataset from *Pik3ca*^*H1047R*^ mutant and control mice.

## Data Availability

The single-cell raw sequencing data and processed counts tables, as well as R files containing raw counts and metadata have been deposited in the Gene Expression Omnibus (accession no. GSE201916). The data on normal dermal LECs is available for searching at https://makinenlab.shinyapps.io/DermaLymphaticEndothelialCells/.
