# Supplementary material for: Immune-interacting lymphatic endothelial subtype at capillary terminals drives lymphatic malformation
Source: J Exp Med. 2023 Jan 23;220(4):e20220741. doi: 10.1084/jem.20220741 (PMC9884640; doi:10.1084/jem.20220741)
Supplement: Table S1 — shows clinical features of patients with LM driven by PIK3CAH1047R mutation. [file JEM_20220741_TableS1.docx]

**Table S1. Clinical features of patients with LM driven by *PIK3CA^H1047R^* mutation**

| **ID** | **Lesion type** | **Gen**  **der** | **Age at biopsy** | **Location** | **Diagnosis** |
| --- | --- | --- | --- | --- | --- |
| Case1  VM168 | Lymphatic malformation (microcystic) | M | 17 yr | Skin (leg) | Epidermis showing acanthosis with hyperkeratosis and papillomatosis; presence of ectatic and congestive dilated vessels containing erythrocytes in superficial dermis; mild chronic perivascular inflammation. Endothelium PECAM1^+^, GLUT1^−^. |
| Case2  VM130 | Mixed veno-lymphatic malformation | F | 13 yr | Nose | Fibrofatty tissue including some skeletal muscle fibers; presence of vessels with irregular morphology, some of them with muscular wall and evidence of lymphoid aggregates. Endothelium PECAM1^+^, PDPN^+/−^, GLUT1^−^. |
| Case3  VM97 | Lymphatic malformation (microcystic) | F | 7 yr | Tongue | Tissue fragments covered by squamous epithelium with hyperplastic changes. Subepithelial stroma contains dilated vascular structures with serous material, lined by endothelium without atypia. Accompanying chronic inflammation. Endothelium PECAM1^+^, PDPN^+^, GLUT1^−^. |

F, female; M, male.
