# Supplementary material for: Immune-interacting lymphatic endothelial subtype at capillary terminals drives lymphatic malformation
Source: J Exp Med. 2023 Jan 23;220(4):e20220741. doi: 10.1084/jem.20220741 (PMC9884640; doi:10.1084/jem.20220741)
Supplement: Table S2 — list antibodies. [file JEM_20220741_TableS2.docx]

**Table S2. List of antibodies**

| **Primary antibody: Immunostaining** | **Company and catalog number** | **Dilution** |
| --- | --- | --- |
| chicken anti-GFP | Abcam, ab13970 | 1/300 |
| goat anti-mouse NRP2 | R&D Systems, AF567 | 1/200 |
| goat anti-mouse VEGFR3 | R&D Systems, AF743 | 1/100 |
| goat anti-human PROX1 | R&D Systems, AF2727 | 1/100 |
| hamster anti-mouse PDPN-AF488 | eBioscience, 53-5381-82 | 1/50 |
| hamster anti-mouse PDPN (clone 8.1.1) | Developmental Studies Hybridoma Bank, 8.1.1-a (Farr et al., 1992) | 1/200 |
| rabbit anti-mouse LYVE1 | Reliatech, 103-PA50AG | 1/500 |
| rabbit anti-mouse Ki67 (clone SP6) | ThermoFischer, MA5-14520 | 1/100 |
| rat anti-mouse PECAM1 | BectonDickinson, 553370 | 1/200 |
| rat anti-mouse EMCN | Santa Cruz Biotechnology, sc-65495 | 1/200 |
| rat anti-mouse CD45 | Abcam, ab25386 | 1/100 |
| rat anti-mouse CD45-FITC | eBioscience, 11-0451 | 1/50 |
| mouse anti-Actin ⍺-Smooth muscle-Cy3 | Sigma, clone 1A4, C6198 | 1/500 |
| rat anti-mouse LYVE1 | R&D Systems, MAB2125 | 1/200 |
| rabbit anti-mouse Collagen IV | Bio-Rad, AbD Serotec, 2150–1470 | 1/1,000 |
| rabbit anti-mouse PTX3 | Thermo Fischer, PA5-38595 | 1/100 |
| rat anti-mouse Ly-6C (HK1.4, APC) | BioLegend, 128015 | 1/50 |
| rat anti-mouse F4/80 (clone Cl:A3-1) | BioRad, MCA497GA | 1/100 |
| hamster anti-mouse PECAM1 (clone 2H8) | Thermo Fischer, MA3105 | 1/300 |
| goat anti-mouse-CCL2 | R&D Systems, AF-479 | 1/100 |
| rabbit anti-human PTX3 | Sigma, HPA069320 | 1/100 |
| mouse anti-human Podoplanin-AF594,  clone D2-40 | BioLegend, 916607 | 1/100 |
|  |  |  |
| **Secondary antibody: Immunostaining** | **Company and catalog number** | **Dilution** |
| donkey anti-rat IgG-Cy3 | JIR, 712-165-153 | 1/300 |
| donkey anti-rat IgG-AF488 | JIR, 712-545-153 | 1/300 |
| donkey anti-rat IgG-AF647 | JIR, 712-605-153 | 1/300 |
| donkey anti-rat-Biotin SP | JIR, 712-065-153 | 1/300 |
| goat anti-Syrian hamster-A5F94 | JIR, 107-585-142 | 1/300 |
| donkey anti-goat-AF647 | JIR, 705-605-147 | 1/300 |
| donkey anti-goat-AF594 | JIR, 705-585-147 | 1/300 |
| donkey anti-rabbit-AF488 | JIR, 711-545-152 | 1/300 |
| donkey anti-rabbit-A647 | JIR, 711-605-152 | 1/300 |
| donkey anti-rabbit-Biotin SP | JIR, 711-065-152 | 1/300 |
| rabbit-anti-hamster-Cy3 | JIR, 307-165-003 | 1/300 |
| donkey anti-rabbit-Cy3 | JIR, 711-166-152 | 1/300 |
|  |  |  |
| **Primary antibody: FACS** | **Company and catalog number** | **Dilution** |
| rat anti-mouse CD16/CD32 | eBioscience, 14-0161 | 1/100 |
| rat anti-mouse PECAM1/CD31  (390, PE-Cyanine7) | eBioscience, 25-0311 | 1/300 |
| hamster anti-mouse PDPN (8.1.1, APC) | eBioscience, 12-7410 | 1/100 |
| hamster anti-mouse PDPN (8.1.1, PE) | eBioscience, 12-5381 | 1/300 |
| rat anti-mouse CD11b  (M1/70, PerCP-Cyanine5.5) | eBioscience, 45-0112 | 1/50 or 1/100 |
| rat anti-mouse CD11b (M1/70, APC) | eBioscience, 17-0112 | 1/100 |
| rat anti-mouse CD11b (M1/70, eF450) | eBioscience, 48-0112 | 1/50 |
| rat anti-mouse CD45  (30-F11, PerCP-Cyanine5.5) | eBioscience, 45-0451 | 1/100 |
| rat anti-mouse CD45 (30-F11, PerCP) | BD Pharmingen, 55-7235 | 1/100 |
| rat anti-mouse CD45 (30-F11, V500) | BD Pharmingen, 56-1487 | 1/100 |
| rat anti-mouse CD45 (30-F11, eF450) | eBioscience, 48-0451 | 1/50 |
| rat anti-mouseF4/80 (BM8, FITC) | BioLegend, 123108 | 1/50 |
| rat anti-mouse Ki67 (SolA15, eFluor 660) | eBioscience, 50-5698 | 1/100 |
| rat anti-mouse TER-119 (TER119, eF450) | eBioscience, 48-5921 | 1/100 |
| rat anti-mouse CD4 (RM4-5, APC) | eBioscience, 17-0042 | 1/100 |
| rat anti-mouse CD3 (17A2, FITC) | eBioscience, 100203 | 1/100 |
| rat anti-mouse B220 (RA3-6B2, eF450) | eBioscience, 48-0452 | 1/100 |
| rat anti-mouse CD8 (53-6.7, SB780) | eBioscience, 78-0081 | 1/100 |
| rat anti-mouse NK (PK136, PE/Cy7) | BioLegend, 108713 | 1/100 |
| rat anti-mouse Ly-6G (1A8, PE/Cy7) | BioLegend, 127617 | 1/100 |
| mouse anti-mouse CD64 (X54-5/7.1, APC) | BioLegend, 139305 | 1/100 |
| mouse anti-mouse Cd11c (N418, PE-Cy7) | eBioscience, 25-0114-81 | 1/100 |
| mouse anti-mouse Ly6C (HK1.4, PerCP.Cy5.5) | eBioscience, 45-5932-80 | 1/100 |
| rat anti-mouse I-A/I-E (M5/114.15.2, Pacific Blue) | BioLegend, 107619 | 1/100 |
| hamster anti-mouse CD11c (N418, BV605) | BioLegend, 117333 | 1/100 |
| rat anti-mouse Cd11b (M1/70, BV605) | BioLegend, 101237 | 1/100 |
| mouse anti-mouse NK-1.1 (S17016D, APC/Cy7) | BioLegend, 156509 | 1/100 |
| rat anti-mouse CD45/B220 (RA3-6B2, APC/Cy7) | BioLegend, 103223 | 1/100 |
| rat anti-mouse CD3 (17A2, APC/Cy7) | BioLegend, 100221 | 1/100 |
| rat anti-mouse Ly6G (1A8, APC/Cy7) | BioLegend, 127623 | 1/100 |
| rat anti-mouse Mer (108928, AF700) | R&D Systems, FAB5912N-100UG | 1/100 |
| rat anti-mouse CCR2 (475301, PE) | R&D Systems, FAB5538P-025 | 1/100 |

JIR, Jackson ImmunoResearch.

References

Farr, A.G., M.L. Berry, A. Kim, A.J. Nelson, M.P. Welch, and A. Aruffo. 1992. Characterization and cloning of a novel glycoprotein expressed by stromal cells in T-dependent areas of peripheral lymphoid tissues. *J. Exp. Med.* 176:1477–1482. 10.1084/jem.176.5.1477
