# Supplementary material for: Immune-interacting lymphatic endothelial subtype at capillary terminals drives lymphatic malformation
Source: J Exp Med. 2023 Jan 23;220(4):e20220741. doi: 10.1084/jem.20220741 (PMC9884640; doi:10.1084/jem.20220741)
Supplement: Table S3 — shows key quality metrics for dermal EC scRNA-seq data. [file JEM_20220741_TableS3.docx]

**Table S3. Key quality metrics for dermal EC scRNA-seq data**

| **Sample ID** | **Mouse genotype** | **Total no. of reads** | **Median no. of reads** | **Reads/**  **cell** | **Genes/**  **cell** |
| --- | --- | --- | --- | --- | --- |
| 895s | Ctrl  C57BL/6J | 110,103,815 | 277,427 | 286,729 | 6,527 |
| 901s | Ctrl  Cre- littermate | 111,465,290 | 280,166 | 290,274 | 5,054 |
| 903s | Ctrl  Cre- littermate | 121,842,883 | 346,507 | 317,299 | 4,294 |
| 891_echo | Mutant  *Pik3ca^H1047R^;Cdh5-CreER^T2^* | 149,568,482 | 381,729 | 389,501 | 6,558 |
| 892s | Mutant  *Pik3ca^H1047R^;Cdh5-CreER^T2^* | 151,102,969 | 367,961 | 393,497 | 5,943 |
| 323s | Mutant  *Pik3ca^H1047R^;Cdh5-CreER^T2^* | 105,188,400 | 245,277 | 273,928 | 6,213 |
| 326s | Mutant  *Pik3ca^H1047R^;Cdh5-CreER^T2^* | 99,738,886 | 254,315 | 259,737 | 4,837 |
| 329s | Mutant  *Pik3ca^H1047R^;Cdh5-CreER^T2^* | 121,560,970 | 301,207 | 316,565 | 4,877 |
| 330s | Mutant  *Pik3ca^H1047R^;Cdh5-CreER^T2^* | 99,829,512 | 196,190 | 259,973 | 5,931 |
| 332s | Mutant  *Pik3ca^H1047R^;Cdh5-CreER^T2^* | 127,499,101 | 292,351 | 332,029 | 5,187 |
